# Computational Modeling of Allosteric Regulation in the Hsp90 Chaperones: A Statistical Ensemble Analysis of Protein Structure Networks and Allosteric Communications

**DOI:** 10.1371/journal.pcbi.1003679

**Published:** 2014-06-12

**Authors:** Kristin Blacklock, Gennady M. Verkhivker

**Affiliations:** 1 School of Computational Sciences and Crean School of Health and Life Sciences, Schmid College of Science and Technology, Chapman University, Orange, California, United States of America; 2 Department of Pharmacology, University of California San Diego, La Jolla, California, United States of America; University of Houston, United States of America

## Abstract

A fundamental role of the Hsp90 chaperone in regulating functional activity of diverse protein clients is essential for the integrity of signaling networks. In this work we have combined biophysical simulations of the Hsp90 crystal structures with the protein structure network analysis to characterize the statistical ensemble of allosteric interaction networks and communication pathways in the Hsp90 chaperones. We have found that principal structurally stable communities could be preserved during dynamic changes in the conformational ensemble. The dominant contribution of the inter-domain rigidity to the interaction networks has emerged as a common factor responsible for the thermodynamic stability of the active chaperone form during the ATPase cycle. Structural stability analysis using force constant profiling of the inter-residue fluctuation distances has identified a network of conserved structurally rigid residues that could serve as global mediating sites of allosteric communication. Mapping of the conformational landscape with the network centrality parameters has demonstrated that stable communities and mediating residues may act concertedly with the shifts in the conformational equilibrium and could describe the majority of functionally significant chaperone residues. The network analysis has revealed a relationship between structural stability, global centrality and functional significance of hotspot residues involved in chaperone regulation. We have found that allosteric interactions in the Hsp90 chaperone may be mediated by modules of structurally stable residues that display high betweenness in the global interaction network. The results of this study have suggested that allosteric interactions in the Hsp90 chaperone may operate via a mechanism that combines rapid and efficient communication by a single optimal pathway of structurally rigid residues and more robust signal transmission using an ensemble of suboptimal multiple communication routes. This may be a universal requirement encoded in protein structures to balance the inherent tension between resilience and efficiency of the residue interaction networks.

## Author Summary

Functional versatility and structural adaptability of the Hsp90 chaperones are regulated by allosteric interactions that allow for diverse functions including modulation of ATP hydrolysis and binding with cochaperones and client proteins. By integrating molecular simulations and network-based approaches we have characterized conformational dynamics and allosteric interactions in different functional forms of Hsp90. The network centrality analysis and structural mapping of allosteric communications have revealed a small-world organization of the interaction network that is mediated by functionally important residues of the Hsp90 activity. We have found that effective allosteric communications in the Hsp90 chaperone may be provided by structurally stable residues that exhibit high centrality properties. Nucleotide-specific rewiring of the network topology and assortative organization of functional residues may protect the active form of the chaperone from random perturbations and detrimental mutations. These results have confirmed that allosteric interactions in the Hsp90 chaperone may be determined by a small-world network of functional residues that can regulate the network efficiency and resiliency by modulating the statistical ensemble of communication pathways in response to functional requirements of the ATPase cycle.

## Introduction

Allosteric regulation is an effective mechanism to control activity and dynamic adaptability of proteins in the cell during signal transduction, metabolism, catalysis, and gene regulation [1]–[4]. Protein allostery is determined by the underlying thermodynamics of a system, where long-range interactions may be determined not only by large structural changes, reflected in the enthalpy contribution, but also by entropy-driven dynamic fluctuations around the average structure [5]–[8]. The statistical model of allosteric regulation is based on the energy landscape theory of protein structure and dynamics, according to which the ensemble of preexisting conformational states and communication pathways can be modulated by allosteric perturbations [9]–[12]. In this model, structural or dynamic perturbation at the effector site could cooperatively shift the relative distribution of the global conformational ensemble and stabilize the allosteric state. Thermodynamics-based approaches associated structural perturbations and allosteric conformational changes with the free energy variations in the conformational ensembles of preexisting states [13]–[15]. Elastic network models of protein dynamics [16], [17] and the normal mode analysis [18], [19] were combined with the theory of signal propagation [20] in the development of statistical models of allosteric communication pathways in proteins [21], [22]. The modern statistical view of protein allostery was further expanded to studies of allosteric protein interactions in signaling pathways and disease states [23]–[25], investigations of disordered proteins [26], modeling of molecular networks [27], and mechanisms of allosteric protein inhibition [28]. Graph-based theoretical approaches can provide a convenient characterization of protein topologies and allow for network-based analyses of protein structure and dynamics. Structure-based network models were successfully used in simulations of protein folding mechanisms [29]–[31], analysis of allosteric communications in proteins [32], [33], and prediction of binding site residues and regulatory sites in protein networks [34]–[37]. These studies suggested that protein structure topologies could produce small-world networks in which a high local connectivity of residue nodes could be balanced by a smaller number of long-range interactions, giving rise to a high degree of interaction cooperativity. Protein structure networks are often described as weighted graphs and use common measures of node centrality [38] to characterize the local connectivity of a particular node (the degree of a node) and global indices of node connectivity (closeness and betweenness). A combination of molecular dynamics (MD) simulations and protein structure network analysis [39]–[44] can characterize topology of allosteric communications in complex molecular assembles by analyzing network centrality and identifying stable communities of interconnected residues. Dynamic-based network approaches employed MD-based contact maps of residue cross-correlations to yield a more accurate description of the network topology and robust description of allosteric communication pathways in tRNA–protein complexes [45], cysteinyl tRNA synthetase [46], imidazole glycerol phosphate synthase [47], [48], thrombin [49], and the M2 muscarinic receptor [50].

**Figure 1:**
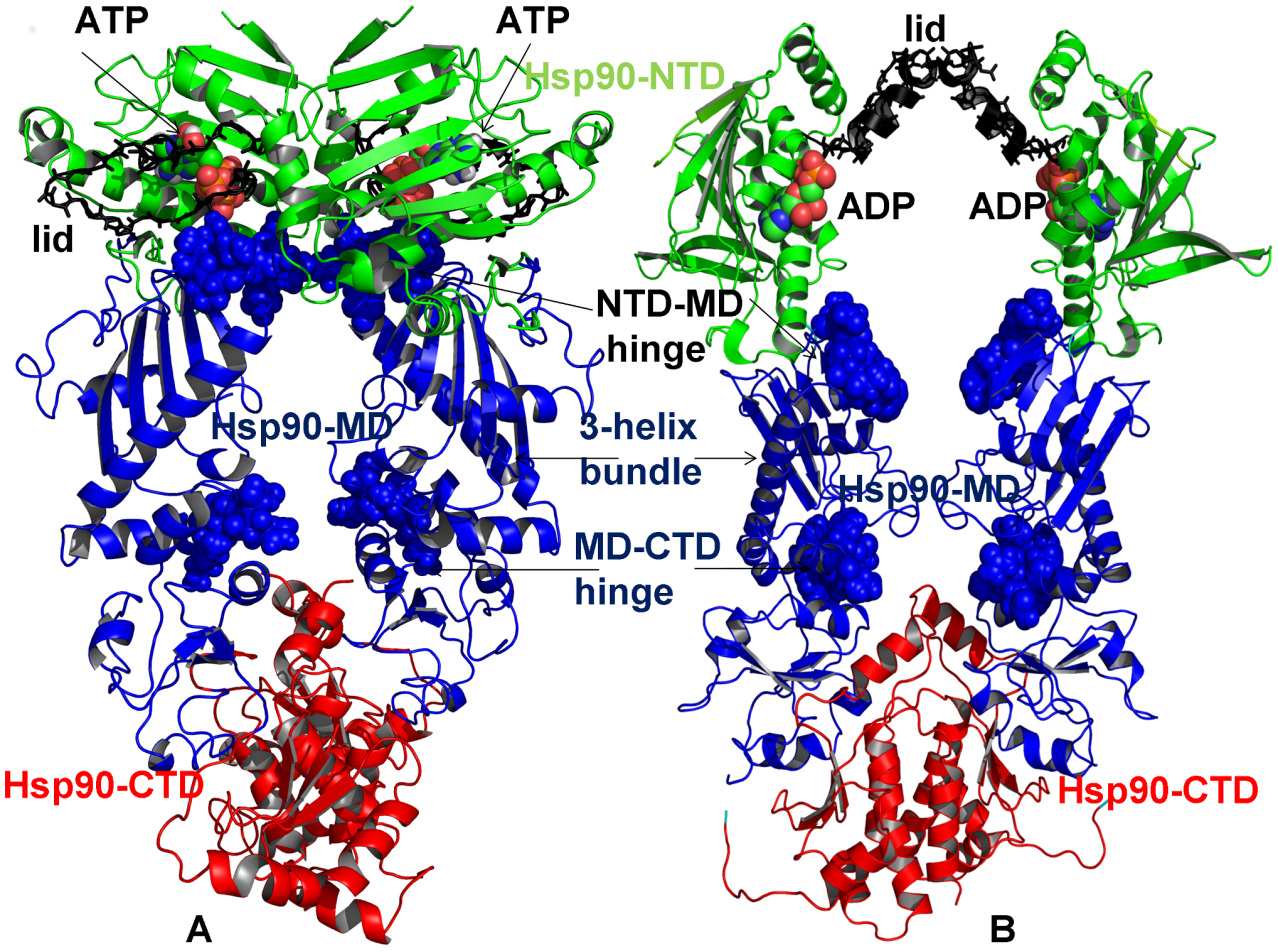
The Architecture and Structural Motifs of the Hsp90 Dimers. The topology and conserved structural regions of the Hsp90 dimer are shown for the crystal structure of yeast ATP-Hsp90 [55] (A), and the crystal structure of ADP-HtpG [56] (B). The structures are shown in a ribbon representation and main structural elements of the Hsp90 dimer are annotated. The Hsp90 domains are colored as follows: Hsp90-NTD (in green), Hsp90-MD (in blue), and Hsp90-CTD (in red). (A) Structural regions of the ATP-Hsp90 dimer. The lid residues 95–123 are highlighted in black sticks. The catalytic loop (371-SEDLPLNLSREMLQQ-385) with a key catalytic residue R-380 is depicted in sticks. The ATP molecules are shown in spheres colored by atoms. The three-helix bundle elements are shown in blue ribbons according to the Hsp90-MD coloration (helix 1: residues 386–408; helix 2: residues 412–431; helix3: residues 435–442) links the inter-domain regions. The inter-domain hinge residues are shown blue spheres. The NTD-MD hinge region (residues 376-LNLSREML-383) also includes catalytic R380. The MD-CTD hinge site includes residues 426-KLGVHE-431. (B) Structural regions of the ADP-HtpG dimer. The functionally important lid region of HtpG (residues 100–126 according to [56]) is highlighted in black sticks as in (A). The ATP molecules are shown in spheres colored by atoms. The three-helix bundle elements are shown in blue ribbons according to the Hsp90-MD coloration (helix 1: residues 336–366 where R336 is the catalytic residue; helix2: residues 368–388; helix 3: residues 393–399). The inter-domain hinge residues are shown in blue spheres. The NTD-MD inter-domain hinge site in HtpG is formed by conserved residues 332-LNVSREIL-339 that contain catalytic R336, and the MD-CTD hinge region includes residues 378-FGLVLKE-384.

The Hsp90 protein (90 kDa heat-shock proteins) is the most abundant molecular chaperone, whose functional versatility and structural adaptability to a broad spectrum of cochaperones and protein clients are regulated by allosteric interactions [51]–[54]. Rapid and efficient transmission of long-range conformational changes plays a vital role in allosteric regulation and may determine the regulatory mechanisms and cellular functions of signaling cascades that are under chaperone's control. The class of Hsp90 chaperones is highly conserved and present in the cytosol of bacteria and all eukaryotes including the eukaryotic cytosolic Hsp90, the bacterial homologue HtpG in *E. coli*, and the endoplasmic reticulum homologue Grp94. The Hsp90 chaperones share a conserved organization of a homodimer in which an N-terminal domain (Hsp90-NTD) is responsible for ATP binding, a middle domain (Hsp90-MD) complements the nucleotide binding site and binds client proteins, and a C-terminal domain (Hsp90-CTD) is involved in dimerization [51]–[54]. Structural studies of Hsp90 in the unbound form and nucleotide-bound complexes from yeast [55], *E. coli* HtpG [56]–[60], and Grp94 homologue [61] determined that Hsp90 could assume a variety of distinct structural forms associated with the ATP binding and hydrolysis (Figure 1). Structural studies supported a mechanism of conformational coupling to the ATPase cycle that is conserved among different species [60] and involves a coordinated transient dimerization of the Hsp90-NTDs and association of the Hsp90-NTD and the Hsp90-MD in the ATP-bound state, but not in the ADP-bound or apo states. Recent biophysical studies using hydrogen exchange mass spectrometry (HX-MS), electron microscopy (EM), small-angle X-ray scattering (SAXS), and single molecule fluorescence resonance energy transfer (FRET) assays confirmed that in the absence of cochaperones and substrate proteins Hsp90 operates in a stochastic conformational cycle associated with ATP binding and hydrolysis [62]–[67]. Remarkably, conformational changes of the Hsp90 chaperone are only weakly coupled to nucleotide binding [64] and the Hsp90-ATPase functional cycle could be primarily determined by thermal fluctuations and spontaneous transitions between different functional forms [65]. A comprehensive reconstitution of the Hsp90 functional cycle [66], [67] demonstrated a controlled progression through distinct intermediate states that can be modulated by conformation-sensitive cochaperones.

The diverse regulatory mechanisms of the Hsp90 machinery are enabled by interactions with a large cohort of cochaperones that can tailor structural and functional plasticity of Hsp90 for demanding needs of client proteins. Allosteric regulation of protein clients by the Hsp90 machine underlies its fundamental role in signal transduction networks associated with protein synthesis, assembly and activation of key signaling proteins driving tumor development and progression. An intriguing aspect of cochaperone function is that cytosolic Hsp90 is the only form of Hsp90 that requires cochaperones for function. The recent structural and functional investigations revealed a diversity of mechanisms by which cochaperones may assist Hsp90 in modulating the progression of the ATPase cycle and recruiting protein clients [68]–[70]. The analysis of cochaperone integration in the ATPase cycle confirmed that the Hsp90 binding with cycle-accelerating cochaperones (Aha1, Cpr6) and cycle-inhibiting cochaperones (p23) can modulate the progression of the conformational transitions by switching the stochastic equilibrium into a more deterministic succession of specific chaperone states [71]. A dynamically controlled binding and release of cochaperones from Hsp90 could modulate the thermodynamics of the ATPase cycle and control conformational transitions required for proper loading and release of the substrate proteins [72]–[74].

In recent years computational studies from several groups have employed structural and dynamic approaches to quantify mechanistic aspects of Hsp90 regulation. Our early studies provided the first atomic resolution models of allosteric regulation in the Hsp90 chaperone and presented evidence of a cross-talk between the N- and C-terminal binding sites of Hsp90 [75], [76]. These computational predictions were subsequently confirmed in the fluorescence-based single molecule assay [63] revealing a strong influence of the ATP binding and hydrolysis in the nucleotide site on the dynamics of C-terminal dimerization. Integration of computational and experimental approaches subsequently led to structural characterization of the binding sites and prediction of allosteric inhibitors targeting the C-terminal binding site in Hsp90 [77]–[80]. A combination of atomistic and coarse-grained approaches with the energy landscape was used to systematically investigate functional dynamics and global motions of the Hsp90 chaperone [81]. This approach identified a network of conserved regions that may be utilized by the molecular chaperone for regulation of the inter-domain communications and control of ATP hydrolysis. All-atom simulations of the Hsp90 crystal structures from different species confirmed that functional dynamics of Hsp90 may be determined by the motion of quasi-rigid domains involving a set of conserved functional residues from the inter-domain hinge sites [82]. Force-distribution analysis based on atomistic simulations of the Hsp90 structures detected allosteric communication pathways connecting the nucleotide-binding site to a distant hinge region and to a putative protein client binding site [83]. Structural and thermodynamic analysis of conformational transitions in Hsp90 using MD simulations described large-scale structural rearrangements and characterized key interactions governing the conformational change [84]. Allosteric regulation of the Hsp90 dynamics and stability by cochaperones and client proteins was pursued in our recent studies [85]–[87]. Functional dynamics analysis of the Hsp90-cochaperone complexes revealed that allosteric interactions in Hsp90 can be selectively modulated by p23 and Aha1 cochaperones that could stabilize specific chaperone conformations [85]. Moreover, the interaction networks in the Hsp90 complexes with p53 [86] and client recruiter cochaperones Cdc37, Sgt1 and Rar1 [87] may produce small-world networks in which highly connected mediating residues at the intermolecular interfaces correspond to the functional hot spots.

In this work, we integrated atomistic simulations of the Hsp90 crystal structures with the structural stability analysis and protein structure network modeling to characterize organization and evolution of the interaction networks and allosteric communications during ATPase-coupled conformational changes of the chaperone. The dynamics-based force constant analysis determines a network of evolutionary conserved and structurally stable residues that can function as mediating sites of allosteric communications in the Hsp90 proteins. Mapping of the principal conformational space of chaperone motions with the network centrality parameters characterizes the dynamic evolution of the conserved interaction networks that can act synchronously with the shifts in the conformational ensemble and determine structural stability of functional states. This analysis can reconcile a broad range of structural and functional experiments by revealing that functional hotspots of the chaperone activity could serve as global mediating centers of structural stability and allosteric communications. We show that dynamic coupling between these functional residues may be important to maintain the robustness of the inter-domain allosteric interactions and modulate the network efficiency in response to functional requirements of the Hsp90-ATPase cycle. The results of this study suggest that a small number of functional residues may be utilized by the chaperone machinery as central regulators of multiple functions, including structural stability, allosteric communications, and binding with cochaperones and client proteins.

## Results/Discussion

### Conformational Dynamics of the Hsp90 Crystal Structures

Multidomain protein structures have complex and variable residue distributions of structural rigidity and flexibility that have evolved to maintain robustness of biological systems to random perturbations and mutations, while allowing for adaptability to new functions. MD simulations of the Hsp90 chaperone in different functional forms were combined with the structural stability analysis to characterize the organization and modularity of structurally rigid and conformationally flexible residues during conformational equilibrium changes. The following specific objectives were addressed in simulations: (a) to determine conserved features and principal differences in the conformational mobility and distribution of structurally stable regions in the Hsp90 chaperone states; (b) to characterize nucleotide-specific changes in the Hsp90 dynamics during the ATPase cycle; and (c) to describe functional coupling between structurally rigid and conformationally flexible regions that may be required for allosteric signaling in the Hsp90 system.

**Figure 2:**
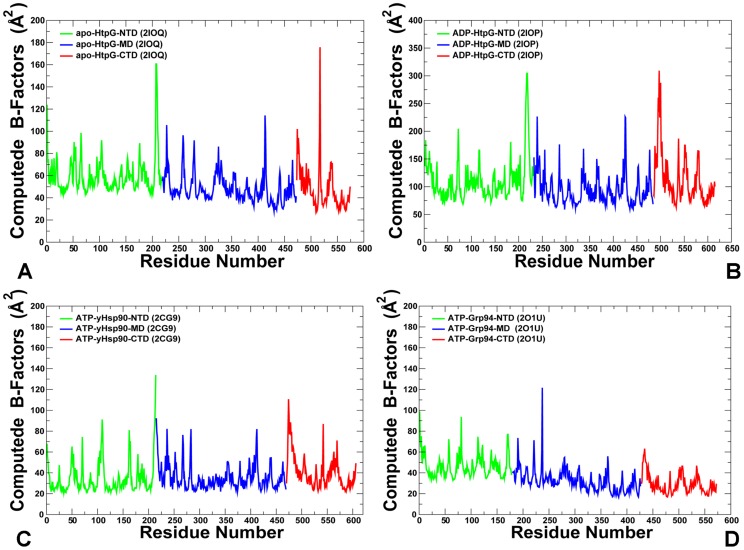
Conformational Dynamics of the Hsp90 Crystal Structures. The computed B-factors are obtained from MD simulations of apo-HtpG [56] (PDB ID 2IOQ) (A); ADP-bound HtpG [56] (PDB ID 2IOP) (B); ATP-bound yeast Hsp90 [55] (PDB ID 2CG9) (C); and ATP-bound Grp94 [61] (PDB ID 2O1U) (D). The NTD residues are shown in green, MD residues are in blue, and CTD residues are in red. The residue-based profiles are based on the consecutive residue numbering adopted from the original crystallographic residue annotation. For clarity, the equilibrium profiles are shown only for one monomer of the homodimer. (A) The fluctuation profile of the apo-HtpG crystal structure has the following residue annotation: NTD (residues 1–219); MD (residues 220–474); CTD (residues 475–577). (B) The profile of the ADP-HtpG crystal structure has the following residue annotation: NTD (residues 1–231); MD (residues 232–486); CTD (residues 487–618). (C) The force constant profile of the yeast ATP-Hsp90 has the following residue annotation: NTD (residues 1–215); MD (residues 216–471); CTD (residues 472–609). (D) The force constant profile of the ATP-Grp94 crystal structure has the following residue annotation: NTD (residues 1–179); MD (residues 180–427); CTD (residues 428–573).

**Figure 3:**
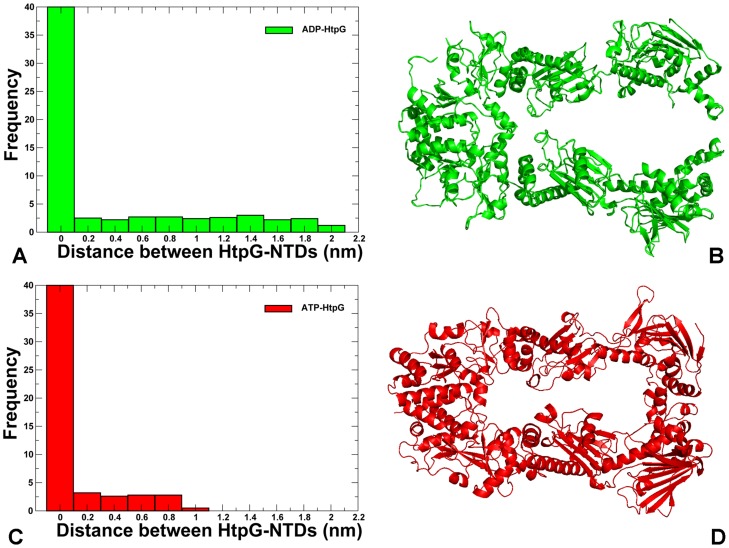
Nucleotide-Dependent Conformational Changes in the HtpG Chaperone. Conformational changes in the ADP-bound HtpG (A,B) and ATP-bound HtpG complexes (C,D) show that HtpG can experience opening and closing motions that are nucleotide-specific. The normalized frequency of the average distance between the HtpG-NTDs is shown for ADP-HtpG as a green-colored histogram (A) and for ATP-HtpG as red-colored histogram (C). The first bar in both histograms is deliberately truncated to make the small fraction of the distribution corresponding to the NTDs separation visible. The average structure of ADP-HtpG is shown in green ribbons (B) and the average conformation of ATP-HtpG is shown in red ribbons (D). The ADP-bound HtpG structure revealed a partial coordinated separation of the NTDs and MDs. The ATP-bound HtpG complex remained in the closed conformation during simulations.

A comparative analysis of the conformational ensembles highlighted fundamental aspects of the Hsp90 dynamics that may be exploited by the chaperone to ensure proper progression of the ATPase cycle. In agreement with the structural studies [56], [57], we found that both apo-HtpG and ADP-bound HtpG states were quite flexible and displayed large thermal fluctuations and the increased conformational mobility as evident from the root mean square fluctuation (RMSF) of the backbone residues and computed B-factors (Figures 2A,B). Although apo-HtpG adopts an open conformation in the crystal and SAXS structures [56], the HtpG-NTDs could experience only local thermal motions and the HtpG-MDs remained closed (Figures 2, S1). Overall, the relative movements between the NTDs and MDs were relatively small in the apo-HtpG structure, which is consistent with the results of FRET experiments [66]. A significantly greater mobility was detected in all domains of the ADP-bound HtpG complex, particularly in the regulatory lid region of the NTD (Figure 2B, S1). Moreover, conformational changes in the ADP-HtpG complex were not limited to the HtpG-NTDs, but also revealed larger protein movements and a partial separation of the HtpG-MDs (Figure 3 A, B). A fairly large conformational change observed in simulations of ADP-HtpG was primarily driven by global motions near the NTD-MD hinge region. In contrast, thermal fluctuations of the ATP-bound HtpG were considerably smaller and the chaperone remained in a closed conformational form throughout the simulation period (Figure 3C,D).

In the ADP-HtpG structure, the nucleotide-specific protection of amide hydrogens was experimentally observed only in the binding site of HtpG-NTD (residues 90–98), whereas some deprotection was widely spread across multiple domains [62]. Our results are consistent with these HX-MS experiments, as high flexibility was observed in the nucleotide-free and ADP-bound HtpG forms, while the addition of ATP could lead to a considerable reduction of thermal motions and stabilization of the closed functional state (Figures 2, 3). Computational predictions are also in an agreement with the atomistic simulations and force distribution analysis of the HtpG chaperone that revealed similar movements of the NTDs and MDs [83]. Noteworthy, the time scale of current MD simulations may not allow to observe a complete conformational equilibrium between well-defined opened and closed ADP-HtpG states, as the equilibrium transitions between these states could be extremely slow [66]. Nonetheless, in agreement with the experiments, simulations showed that the ADP and ATP nucleotide binding can differentially modulate equilibrium between open and closed dimer conformations of HtpG. Moreover, the observed conformational flexibility and weakened inter-domain interactions in the ADP-HtpG chaperone are consistent with the fluorescence spectroscopy [57] and HX-MS dynamic studies of HtpG [62], in which the ADP-bound HtpG form could not be detected as a stable intermediate in solution and was proposed to exist only transiently.

MD simulations of the ATP-bound yeast Hsp90 demonstrated small thermal fluctuations occurring near the crystallographic conformation, where the NTDs and CTDs remained in a closed structural arrangement (Figure 2C). These results are consistent with the experimental data and our previous studies [76], [81] confirming that structural stability of the inter-domain interactions is a fundamental characteristic of the ATP-bound Hsp90 complex. A different distribution of structurally stable regions was observed in simulations of the ATP-bound Grp94 homologue (Figure 2D). While the Grp94-NTDs appeared to be mobile, the thermal movements of the MD and CTD regions were fairly small, which is consistent with the experimental data, since nucleotide binding had a small effect on conformational rearrangements in the Grp94 chaperone, yet even a partial truncation of the peripheral CTD regions may lead to a considerable loss of the hydrolysis activity [61].

### The Ensemble-Based Structural Stability Analysis

The results of MD simulations were utilized in the force constant analysis of structural stability performed for different functional forms of Hsp90 [88], [89]. In this approach, the fluctuations of the mean distance between each residue and the rest of the protein are translated into force constants that measure the energy cost of the residue displacement during equilibrium simulations. The residues with high force constants are typically structurally rigid and abrupt changes between maxima and minima in the distributions may signal the emergence of boundaries between structurally rigid modules and flexible regions, often pointing to the inter-domain hinge sites. The hypothesis tested in this analysis is that functional hotspots of the chaperone activity could be involved in mediating structural stability and allosteric communications in Hsp90 by bridging high and low stability regions near the inter-domain hinge sites. In this formulation, structural stability of the inter-domain regions may be an important determinant that controls global chaperone movements and ensures normal progression of the ATPase cycle. The residue-based force constant profiles can be also associated with allosteric communication capabilities. We previously formulated a model that defined communication propensities of protein residues by relating the mean square fluctuations between a pair of residues to their commute time [85]. According to this model, structurally stable residues with high force constants may display small fluctuations in their distances to other residues and often correspond to effectively communicating rigid sites. We analyzed the organization of structurally stable residue modules in the Hsp90 structures to identify and characterize key functionally residues that may contribute to structural stability, allosteric communications, and chaperone activity. The peaks of the force constant distributions were also mapped onto conformational mobility profiles in the essential space of low frequency motions in order to determine if structurally stable regions can act synchronously with the shifts in the conformational ensemble.

**Figure 4:**
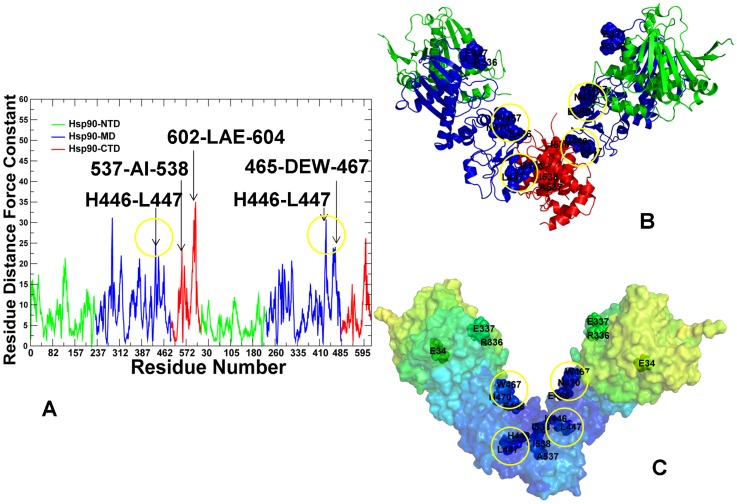
The Force Constant Stability Analysis of the apo-HtpG Chaperone. (A) The residue-based force constant profile of apo-HtpG. The NTD residues are in green, MD residues are in blue, and CTD residues are in red. The residue-based dynamic profiles are annotated using the residue numbering in the original crystal structure [56]. The peaks of the force constant profiles corresponding to functionally important residues are indicated by arrows and annotated. Functional residues corresponding to the peaks in the force constant distribution are mapped onto the domain-colored crystal structure of apo-HtpG (B) and onto the functional dynamics profile of apo-HtpG (C). The functional dynamics profile is obtained using PCA of the MD-based conformational ensembles averaged over three lowest frequency modes. The color gradient in from blue to red indicates the decreasing structural stability (or increasing conformational mobility) of protein residues. Functional residues are annotated and shown in spheres, colored according to their domains in (B) and according to the level of rigidity (flexibility) in the functional dynamics profiles (C).

**Figure 5:**
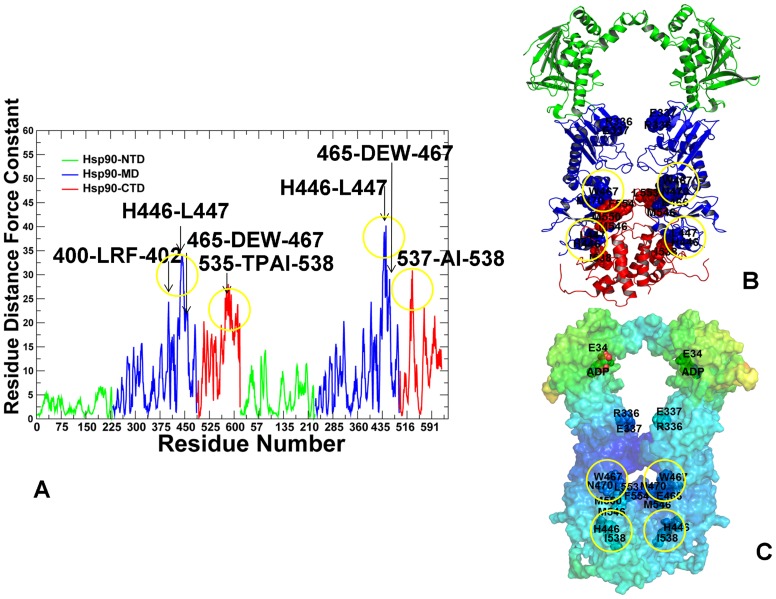
The Force Constant Stability Analysis of the ADP-bound HtpG Chaperone. (A) The residue-based force constant profile of the ADP-bound HtpG structure. The NTD residues are in green, MD residues are in blue, and CTD residues are in red. The residue-based dynamic profiles are annotated using the residue numbering in the original crystal structure [56]. The peaks of the force constant profiles corresponding to functionally important residues are indicated by arrows and annotated. Functional residues corresponding to the peaks in the force constant distribution are mapped onto the domain-colored crystal structure of ADP-HtpG (B) and onto the functional dynamics profile of ADP-HtpG (C). Functional residues are annotated and shown in spheres and colored as in Figure 4.

The force constant profiles for the HtpG structures were characterized by a moderate number of high peaks and mostly reflected conformationally flexible nature of the apo (Figure 4) and ADP-bound HtpG states (Figure 5). Moreover, the distribution peaks were narrow and often corresponded to single rigid residues rather than consolidated structurally rigid modules. The NTD-MD hinge site in HtpG is formed by conserved residues 332-LNVSREIL-339 that include catalytic R336, while the MD-CTD hinge region consists of residues 378-FGLVLKE-384 and 393-QEAIAKL-399 (Figure 1). Importantly, the identified structurally stable regions mapped onto a number of well-characterized functional residues of the HtpG homologue, including hinge sites at the inter-domain regions (Figure 4B, 5B). Notably, the force constant peaks for apo-HtpG (Figure 4) and ADP-HtpG (Figure 5) corresponded to functionally critical H446 and L447 residues that contribute to stabilization of the MD-CTD interfacial regions. SAXS experiments have identified that pH-dependent conformational equilibrium for apo-HtpG that can be effectively modulated by mutations of a single residue H446 [58]. According to this study, the H446K mutant can shift conformational preferences towards a more compact Grp94-like state, which is adopted by HtpG-WT at low pH, whereas H446E mutation can push the equilibrium to a more extended conformation that is assumed by HtpG-WT at high pH. The force constant profiles of apo-HtpG and ADP-HtpG also exhibited peaks in the functionally important region 465-DEWMMN-470 (Figures 4, 5). This motif bridges the HtpG-MD and HtpG-CTD regions and contains functionally important residues E466, W467, and N470 whose mutants could significantly compromise the ATPase activity and diminish the ability of the chaperone to activate a diverse range of substrate proteins [74]. Another common structurally rigid site 535-TPAIV-539 is situated in the HtpG-CTD and contains a conserved regulatory switch I538 [90]. Hence, we found that functional chaperone residues could often correspond to the force constant profile peaks and occupy the inter-domain regions bordering high and low stability regions.

**Figure 6:**
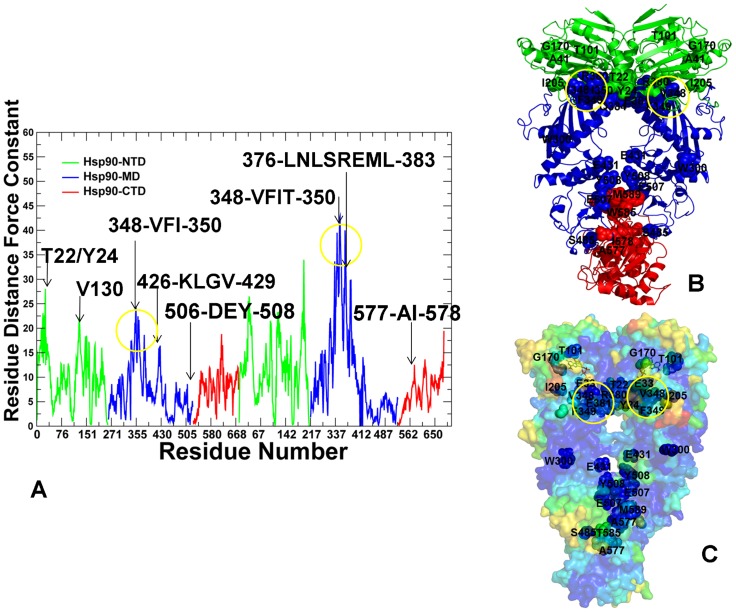
The Force Constant Stability Analysis of the ATP-bound yeast Hsp90 Chaperone. (A) The residue-based force constant profile of the ATP-bound yeast Hsp90 structure. The NTD residues are in green, MD residues are in blue, and CTD residues are in red. The residue-based dynamic profiles are annotated using the residue numbering in the original crystal structure [55]. The peaks of the force constant profiles corresponding to functionally important residues are indicated by arrows and annotated. Functional residues corresponding to the peaks in the force constant distribution are mapped onto the domain-colored crystal structure of the ATP-bound yeast Hsp90 (B) and onto the functional dynamics profile of the ATP-bound yeast Hsp90 (C). Functional residues are annotated and shown in spheres and colored as in Figure 4.

The force constant profiling of the ATP-bound yeast Hsp90 suggested a considerable stabilization of the NTD and MD residues, especially the inter-domain interfaces, as evident from the reduced number of conformationally flexible (low force constant) residues in these regions (Figure 6). Importantly, this profile revealed the emergence of broad peaks, reflecting consolidation of structurally stable residues into interconnected rigid modules. The force constant peaks in the Hsp90-NTDs corresponded to functional residues T22, Y24, and A41 that contribute to stabilization of the inter-monomer interface and determine a closed structural arrangement of the ATP-Hsp90 dimer [91], [92]. Mutations of the Hsp90-NTD residues (T22, Y24, and T101) could modulate the ATPase activity and reduce client protein activation. Interestingly, a single mutation T101I could disfavor the ATP-induced conformational switch in Hsp90 and reinforce interactions with the Cdc37 cochaperone, whereas T22I can induce this conformational switch and has the opposite effect on binding with Cdc37 [92], [93]. Mutations of Y24 and T22 to glutamic acid can also markedly impair Hsp90-ATPase activity and the produced chaperone defect in cells could be even more severe [93]. The broad peaks in the distribution also corresponded to structurally stable modules from the Hsp90-MD (residues 348-VFITDE-353, 376-LNLSREML-383, and 426-KLGV-429). These interaction clusters include functionally important residues near the NTD-MD interface region (V348, F349, I350) that are involved in stabilization of the inter-domain interactions required for the formation of the active dimer. Structural and functional analysis of yeast Hsp90 has demonstrated that F349A and F349Q mutations could severely reduce ATPase activity in vitro and impair client activation function [94]. Another important peak in the force constant distribution corresponded to the functional loop 376-LNLSREML-383 that harbors the critical catalytic residue Arg-380 and hydrophobic residues L376, L378 (Figure 6C). These functional residues act synergistically using a combination of the charged interactions and cross-monomer hydrophobic interactions to stabilize the closed and catalytically active dimer conformation [95]. A sharp peak in the force constant profile was also associated with the stable motif 426-KLGV-429 near the MD-CTD inter-domain hinge (Figure 6). This motif belongs to the helix 2 (residues 412–431) of the three-helix bundle (Figure 1) that displayed the increased protection in HX-MS experiments [62]. Smaller peaks were also observed for functional residues (E507, Y508, and T511) whose mutations can reduce chaperone activity in vivo and adversely affect binding of client proteins [74]. These findings reinforced the importance of the inter-domain hinge regions as stable functional modules that can control conformational transitions during the Hsp90-ATPase cycle.

Structural studies of the Grp94 homologue in complexes with AMP-PNP and ADP [61] have revealed a partially closed conformation that is catalytically silent and conformationally insensitive to the identity of the bound nucleotide. The rigidity profile of the ATP-bound Grp94 (Figure S2) revealed low force constant values (high conformational flexibility) for the NTD residues and the NTD-MD region of the second monomer. The weakened inter-domain interactions in ATP-Grp94 may be responsible for the lack of productive dimerization interactions between the two NTDs and explain why ligand-induced conformational changes in Grp94 are typically limited only to the N-termini regions [61]. We also observed that the Grp94-NTD residues near the inter-domain boundaries may exhibit a different level of flexibility in two monomers. At the same time, high force constant values were seen for residues located near the MD-CTD interface. The force constant peaks in the Hsp90-MD corresponded to mostly hydrophobic residue clusters 494-LGVIED-499, 425-MMPKYL-430, and 658-MERIM-662 (Figure S2). Consistent with these results, structural experiments indicated that a conserved M658-M662 pair could present a client-binding site in Grp94 [61]. Conformational flexibility of the Grp94-NTDs may partly explain why functional residues, which are located at the NTD-MD interface, could become immune to mutations. Indeed, mutations of the Grp94 residues R448 (corresponding to R380 in yeast), Q452 (Q384 in yeast) and hydrophobic patch 416-VF-417 (348-VF-349 residues in yeast Hsp90) do not induce loss of ATPase activity [96], [97].

In contrast, some of these residues in yeast ATP-Hsp90 (R380, 348-VF-349) can act as ATP-sensitive stabilizers of the inter-domain interactions and dimerization [98], [99]. Our analysis captured these important differences between yeast Hsp90 and Grp94, as the corresponding residues exhibited high force constants (high structural stability) in yeast Hsp90 (Figure 6), but displayed moderated force constant values and conformational flexibility in Grp94 (Figure S2). In summary, the force constant analysis of structural stability recapitulated a broad range of experimental observations by linking structural stability and communication propensity of protein residues with their importance in the ATPase cycle and chaperone activity.

### Nucleotide-Based Modulation of Structural Stability and Solvent Protection in the Hsp90 Crystal Structures: Computation and Experiment

We compared our computational results with the HX-MS and fluorescence spectroscopy analysis of the conformational dynamics of HtpG and yeast Hsp90 in solution [62]. In these experiments, the addition of ADP induced a small but broadly distributed amide hydrogen deprotection and a greater flexibility in the HtpG-NTD lid segment (residues 108–119), the HtpG-MD (residues 279–299 and 401–416) and most of the HtpG-CTD regions. Consistent with these experiments, our analysis revealed that force constant values for these residues were reduced in the ADP-HtpG and corresponded to the distribution minima, thus pointing to the conformationally flexible regions (Figure 5A). A considerably stronger effect was experimentally observed in the ATP-HtpG complex, where amide hydrogen protection and the increased structural rigidity was detected in the HtpG-NTD (residues 2–19, 21–31, 90–98, 121–127, 192–206) and HtpG-MD regions (residues 319–334, and 336–359) [62]. Experimental measurements are typically confined to the backbone amide protons, which are involved in hydrogen bonding for the maintenance of secondary structure, yet amides buried from the solvent but not hydrogen bonded may also have very slow exchange rates. The experimentally observed nucleotide-dependent changes in the hydrogen–deuterium exchange for the Hsp90 structures may thus depend on both structural rigidity and solvent accessibility. To facilitate a quantitative comparison with the experimental HX-MS data, we supplemented the force constant stability predictions with the ensemble-based analysis of solvent accessibility. In order to differentiate between buried residues located near the protein surface and inside the protein core, we used a computational procedure for calculating the depth of a residue from the protein surface [100]. A differential plot of the residue depth profiles was computed for apo-HtpG and ATP-HtpG using MD-based equilibrium ensembles (Figure S3A). Consistent with the HX-MS data, this analysis not only detected the increased and broadly distributed protection from solvent in the ATP-HtpG structure, but also closely mapped these changes onto the respective NTD (residues 90–96,121–127) and MD regions (residues 319–359). Nucleotide-induced protection in the ATP-bound HtpG reflected a shift in the conformational equilibrium towards a closed conformational form that was observed in simulations (Figure 3C, D). Our analysis also indicated that the improved protection of the NTD-MD hinge site (residues 332-LNVSREIL-339) may be important in modulating allosteric communications in the closed conformation of the ATP-HtpG complex. In agreement with the experiments, these results indicated that conformational equilibrium changes in HtpG may be strongly coupled to the nucleotide turnover by following a mechanical ratchet mechanism [66]. In this mechanism, the HtpG chaperone can experience opening and closure of the HtpG-NTDs over a large distance via thermal fluctuations, whereas ATP binding can selectively stabilize a closed conformational form of the chaperone.

In yeast Hsp90, the effect of ATP-induced protection was especially pronounced in the nucleotide-bound lid segment, while small protection was broadly distributed in the Hsp90-MDs and Hsp90-CTDs. The hydrogen exchange profile in the yeast ATP-Hsp90 pointed to the increased rigidity in the nucleotide-interacting lid residues 91–105 and 118–124, the Hsp90-MD (residues 341–349, 418–435, and 477–492) and in the Hsp90-CTD residues 622–642 [62]. The force constant analysis of ATP-Hsp90 predicted structural stabilization in these regions, particularly emphasizing a critical role of the NTD lid motif and the inter-domain MD residues forming modules of strongly interacting residues (Figure 6). To further quantify a comparison with the HX-MS data, we also computed the ensemble-averaged residue depth profiles for apo and ATP-bound yeast Hsp90 (Figure S2B). This analysis reproduced a moderate but broadly distributed effect of the nucleotide binding. Consistent with the HX-MS data, the increased amide protection was properly mapped onto the lid segment (residues 91–105 and 118–124), the Hsp90-MD regions (residues 380–435,470–492) and the Hsp90-CTD segments (residues 540–600) (Figure S3B). The increased protection was also detected for the hinge regions (residues 376-LNLSREML-383, 426-KLGVHE-431). Computational predictions using both the force constant analysis and the residue depth profiling pointed to the ATP-induced stabilization of the inter-domain regions. Noteworthy, small deprotection peaks corresponded to single flexible residues distributed across all domains, while a consistent pattern of the enhanced protection in functional regions may be statistically significant and reflect the effect of ATP-induced stabilization of a closed dimerized state. Our results are consistent with the experimental findings suggesting that conformational changes in yeast Hsp90 are less sensitive to the nucleotide binding and may be driven by slow thermal fluctuations between the open and closed states [66]. The recruitment of cochaperones into Hsp90 can turn the stochastic conformational changes in yeast Hsp90 into a successive progression of functional states by precisely tuning structural stability of the nucleotide-bound complexes during the ATPase cycle [71].

### Protein Network Analysis of the Hsp90 Crystal Structures: Conservation of Structurally Stable Interaction Networks

**Figure 7:**
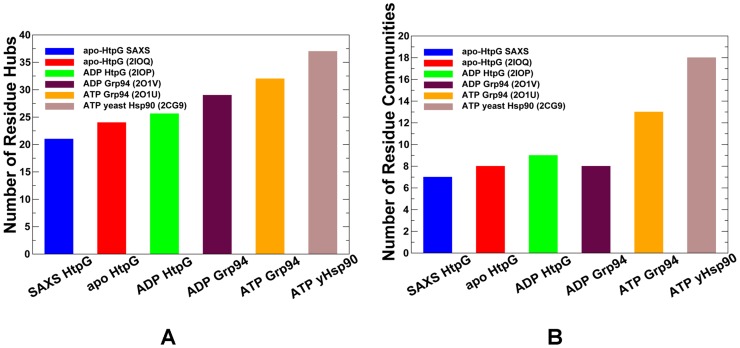
The Network Analysis of the Hsp90 Crystal Structures. The distribution of hubs (A) and communities (B) in different functional states of Hsp90. The distributions of protein structure network parameters are obtained by averaging computations over MD simulation trajectories. The analysis is based on structurally stable residue interaction networks that remained intact in more than 75% of the simulation snapshots. The distributions are shown for the open solution conformation of HtpG obtained from SAXS studies [57], [58] (in blue); an apo form of HtpG (PDB ID 2IOQ) [56] (in red); an ADP-bound form of HtpG (PDB ID 2IOP) [56] (in green); an ADP-bound form of the Grp94 homologue (PDB ID 2O1V) [61] (in maroon); an ATP-bound form of the Grp94 homologue (PDB ID 2O1U) [61] (in orange); and an ATP-bound conformation of yeast Hsp90 (PDB ID 2CG9) [55] (in brown).

**Figure 8:**
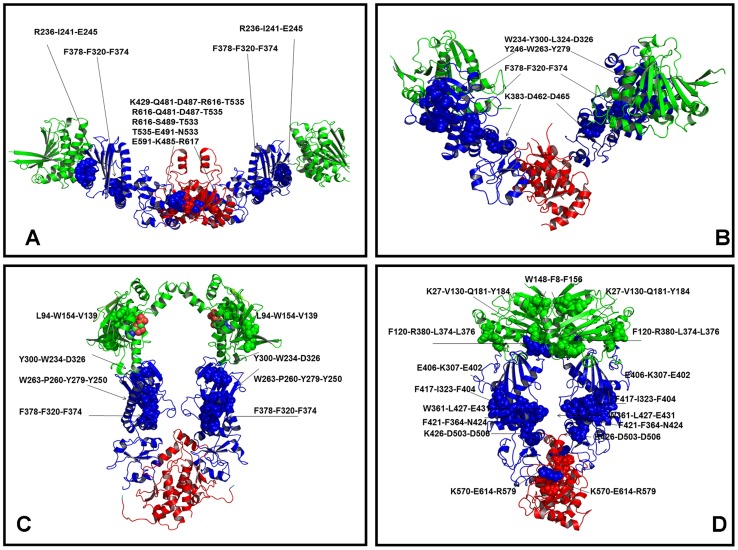
Structural Maps of Stable Interaction Communities in the Hsp90 Structures. The distribution of structurally stable interaction communities is shown for the SAXS structure of HtpG (A); the crystal structure of apo HtpG (B); the crystal structure of ADP-HtpG (C); and the crystal structure of yeast ATP-Hsp90 (D). The Hsp90 structures are shown in a ribbon representation and colored according to their domain nomenclature: Hsp90-NTD (in green), Hsp90-MD (in blue), and Hsp90-CTD (in red). The Hsp90 residues that constitute structural communities are shown in spheres and colored according to their respective domains. The residue-based annotation of stable communities is provided and structural positions of communities are indicated by arrows. Structural mapping of stable communities in the solution HtpG structure (A) and ADP-HtpG structure (C) shows a partly disjointed pattern of the interaction networks that are mostly confined within the individual domains. The ATP-bound dimer of yeast Hsp90 has a dense interaction network that includes both the inter-domain and the inter-monomer communities. The Pymol program was used for visualization of the Hsp90 structures (The PyMOL Molecular Graphics System, Version 1.2r3pre, Schrödinger, LLC).

We also employed a structure-based network analysis of the Hsp90 chaperones to characterize the organization of local hubs and stable interaction communities as a simple and robust metric for evaluation of structural stability. Protein structure networks were constructed by incorporating both the topology-based residue connectivity and contact maps of residues cross-correlations obtained from MD simulations of the Hsp90 crystal structures. Mapping of stable interaction communities onto the conformational dynamics profiles of the Hsp90 structures was done to address the following specific objectives: (a) to determine structurally conserved communities shared by different functional forms of Hsp90; and (b) to characterize the reorganization of residue interaction networks during conformational equilibrium changes. Despite different domain organization within each monomer and significant structural rearrangements of the Hsp90 dimer, the average number of hubs (Figure 7A) and stable interaction communities (Figure 7B) in the Hsp90 structures was fairly similar. The distribution of structurally stable networks was consistent with the results of SAXS and EM experiments, supporting the notion that small free energy changes between functional states may be a fundamental characteristic of the conformational equilibrium in Hsp90 [57]–[60]. The number of stable communities (Figure 7B) moderately increased in the ATP-Grp94 complex and became appreciably larger only in the yeast ATP-Hsp90 structure. To characterize the stability of the interaction networks during conformational equilibrium changes, we mapped structural communities onto the dynamics profiles of the Hsp90 crystal structures in the space of principal modes (Figure S4). The communities were primarily aligned with structurally rigid regions in the population distribution profiles, thus suggesting that stable interaction networks may act concertedly with the shifts in the conformational equilibrium. We systematically examined the distribution of structurally stable communities in different functional forms of Hsp90. A scattered arrangement of local communities could be seen in the SAXS structure of apo-HtpG, with multiple communities formed by the MD and CTD residues (Figure 8A). In the apo-HtpG structure, the interaction communities were primarily formed by the Hsp90-MD residues, but were also found in the Hsp90-NTD and Hsp90-CTD regions (Figure 8B). The presence of stable communities (F378-F320-F374, K383-D462-D465) near the inter-domain regions could ensure structural stability of the hinge sites that may be essential to initiate the ATPase cycle and coordinate progression of conformational changes. Despite significant differences in the domain assembly, the local communities (W234-Y300-L324-D326) and (F378-F320-F374) were shared by the HtpG states, even though the level of exposure of the hydrophobic residues in the central cleft is drastically different in these structures. We noticed that the distribution of stable communities in the ADP-HtpG form (Figure 8C) was characterized by a partly disjointed pattern of the interaction networks that were mostly confined within the individual domains. Accordingly, the lack of the inter-domain communities may indicate the weakened inter-domain interactions that could promote the increased conformational flexibility and adversely affect allosteric signaling in the ADP-bound HtpG structure.

The central characteristic of the ATP-bound state is the emergence of both the inter-domain and the inter-monomer communities. Moreover, the reorganization of structurally stable communities in ATP-Hsp90 may lead to the aggregation of small communities into larger interaction networks. A dense interaction network could rigidify the active dimer and effectively immobilize hinge sites (Figure 8D). The first hinge site (residues 376-LNLSREML-383) containing catalytic Arg-380 is located at the NTD-MD interface, while the second inter-domain hinge (residues 426-KLGVHE-431) resides at the MTD-CTD boundary. One of the inter-domain communities was formed by the Hsp90-MD residues (L376, L378, R380) that interact with the Hsp90-NTD residue F120 to bridge both the inter-domain and the inter-monomer interactions in the Hsp90 active dimer (Figure 8D). The important role of this interaction network (F120-R380-L376-L378) is consistent with a synergistic role of the contributing residues in ATP hydrolysis, inter-domain communication, and client binding [98], [99]. Additionally, the contribution of these hydrophobic interactions can be enhanced through interactions of the Hsp90-MD residues (L376, L378, and R380) with the Hsp90-NTD residues of the other monomer (T22, V23, and Y24) [99]. These residues may act synergistically to stabilize the hydrolysis-competent conformation of Hsp90. We also found that a group of partially overlapping local communities (W361-L427-E431), (F421-F364-N424) and (K426-D503-D506) can contribute to stabilization of the MD-CTD interface. Interestingly, one of these networks (K426-D503-D506) is located in the immediate vicinity of functional residues (E507, Y508, T511, and W585), whose mutations could affect viability and chaperone activity in vivo [74]. It is possible that structural stability of functional residues E507, Y508, T511 may be protected by contacts with the adjacent inter-domain communities. Mutation-induced structural perturbations caused by detrimental chaperone variants (E507R, Y508R and W585T) may exert their adverse effect by compromising structural stability of the inter-domain community (K426-D503-D506). The dominant contribution of the inter-domain interaction networks may be important for thermodynamic stabilization of the active Hsp90 dimer that is required to modulate normal progression of the ATPase cycle.

### Small-World Interaction Networks in the Hsp90 Chaperone: The Local Residue Hubs

**Figure 9:**
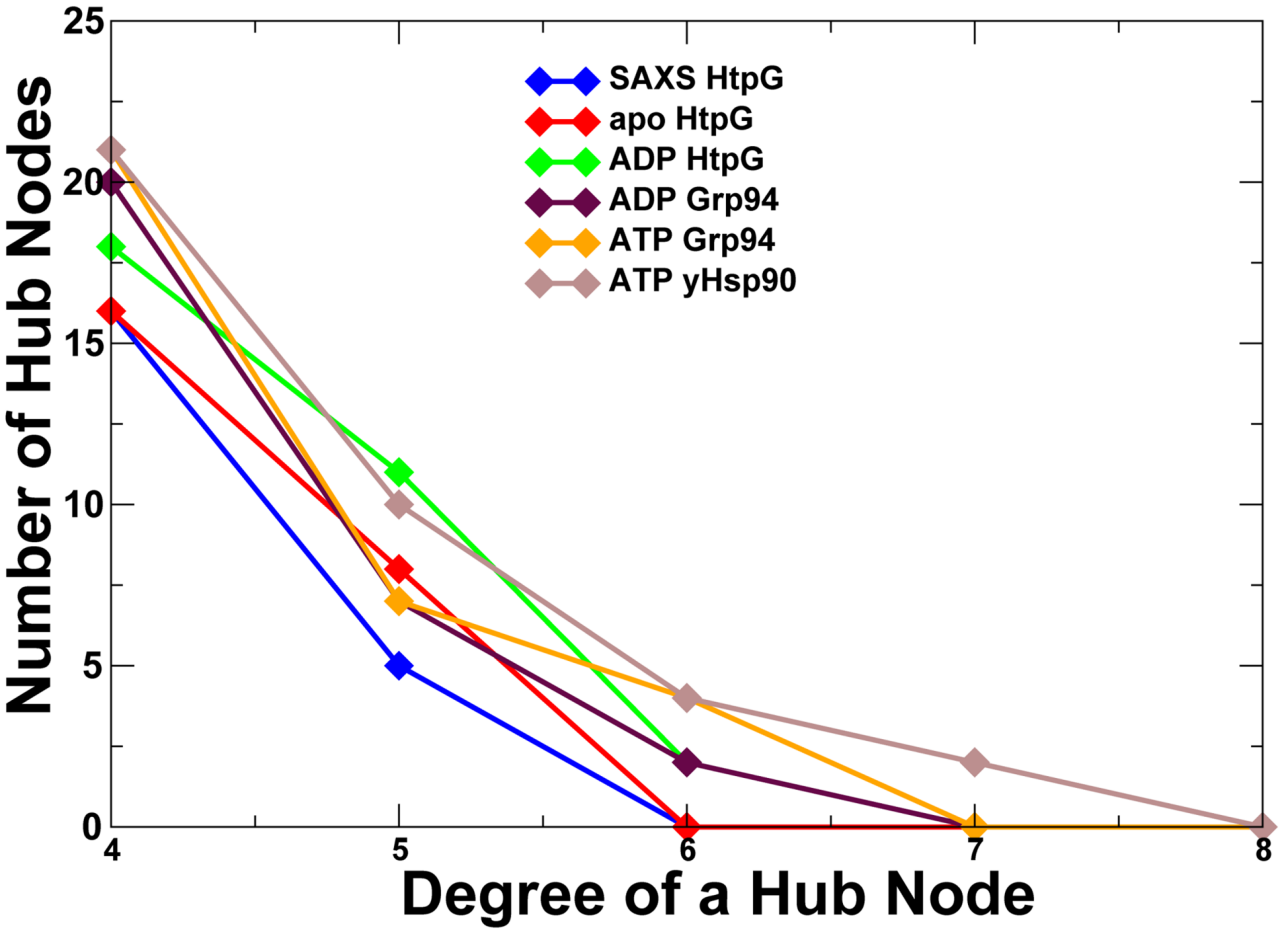
The Degree Distribution of Residue Hubs in the Hsp90 Structures. The number of hub nodes as a function of the degree of a hub is shown for the solution HtpG structure (in blue); the apo-HtpG structure (in red); the ADP-HtpG structure (in green); the ADP-Grp94 structure (in maroon); the ATP-Grp94 structure (in orange); and the yeast ATP-Hsp90 structure (in brown). The coloring annotation of the distribution is consistent with the annotation of residue hubs in Figure 7.

By mapping network centrality parameters onto MD-derived functional dynamics profiles of the Hsp90 structures, we modeled the organization and evolution of the residue interaction networks during conformational equilibrium changes. First, we explored the simplest centrality measure, the degree of a node, which is defined as the number of interacting residues that a particular residue node is connected to. The degree of a node is also referred as a radial measure of network centrality [101]. The network organization of protein structures may be determined by the average degree correlation between nodes, so that complex networks may be either disassortative, where the links between nodes with similar networking parameters are prevented, or assortative, where these links are enhanced [102]–[105]. While a disassortative organization allows for rapid signal transmission between segregated modules, but may produce more vulnerable to random attacks networks, assortative networks may sacrifice the efficiency of long-range communication to achieve a greater resilience against random perturbations. We computed the spatial distribution of local residue hubs to determine if highly connected hub residues in the Hsp90 structures may be connected to other hubs with similar networking characteristics, thus exhibiting signs of assortative mixing. We observed that the degree distributions of hub residues in different structural forms of Hsp90 were quite similar (Figure 9). The number of hub nodes exponentially decayed as the degree of a hub node increased. In agreement with network-based studies of protein structures [35], we found that the distribution may follow the Poisson model [106]–[111] and nodes that significantly deviated from the average degree were fairly rare. In a scale-free network organization, a node is more likely to form an edge with another node that has a considerably higher than average number of attachments or the shortest path length to a given node. Protein structures have a considerably smaller number of highly connected hubs as compared to most self-organized cellular networks due to a limited interacting capacity of a given residue within a given structural fold.

We analyzed structural distributions of hub residues (Figures S5, S6) by focusing on highly connected hubs with the number of connected residues exceeding the threshold of four. The local hubs in the HtpG structures may have on average the fewer interacting neighbors than the hub residues in the ATP-Grp94 and yeast ATP-Hsp90 structures (Figures S5, S6). This is consistent with the inherent conformational flexibility of the apo and ADP-bound HtpG states, as only a relatively small number of residues could establish a stable interaction environment with multiple residues. In contrast, the number of local hubs was noticeably greater in the ATP-Grp94 and the ATP-Hsp90 structures (Figure S6). In the ATP-Hsp90 dimer, a number of highly connected hubs were locally connected with other hubs of a similar degree, suggesting that small-world interaction networks in the active form of Hsp90 may display assortative features. The interactions between local hubs with similar node degree are often referred as the “rich-club” phenomenon [112], [113]. In this mechanism, central hubs may be supported by neighboring hub residues that provide functional redundancy and robustness to hub failures caused by random mutations. This phenomenon may also reflect the increased level of cooperativity in the functional chaperone form, in which hub residues from different domains could interact and act concertedly, thus “coordinating” their activities during the Hsp90-ATPase cycle.

### Allosteric Communications in the Hsp90 Chaperone: The Network Centrality Analysis

We also explored the global network parameter, betweenness, to model allosteric communication pathways and identify a network of critical mediating nodes involved in long-range signaling. The betweenness of a node is defined as the number of shortest paths that pass through that node in the network, representing a global medial measure of the node contribution to the communication flow within the network. Betweenness indicates how frequently a node lies along geodesic pathways of other nodes in the network and is an inherently asymmetric measure. The role of chaperone residues in allosteric signaling was evaluated by computing the ensemble-based betweenness index and considering residue peaks in the centrality profiles as potential mediating sites of long-range communication. We proposed that effective allosteric communications in the Hsp90 chaperone can be primarily provided by structurally stable residues that exhibit a significantly higher betweenness as compared to the network average. Using network centrality analysis, we investigated whether allosteric communications in the Hsp90 chaperone could operate via a predominantly single “rigidity propagation path” or may follow a more general mechanism, in which structurally rigid and flexible residues would act cooperatively to form an ensemble of multiple communication pathways.

**Figure 10:**
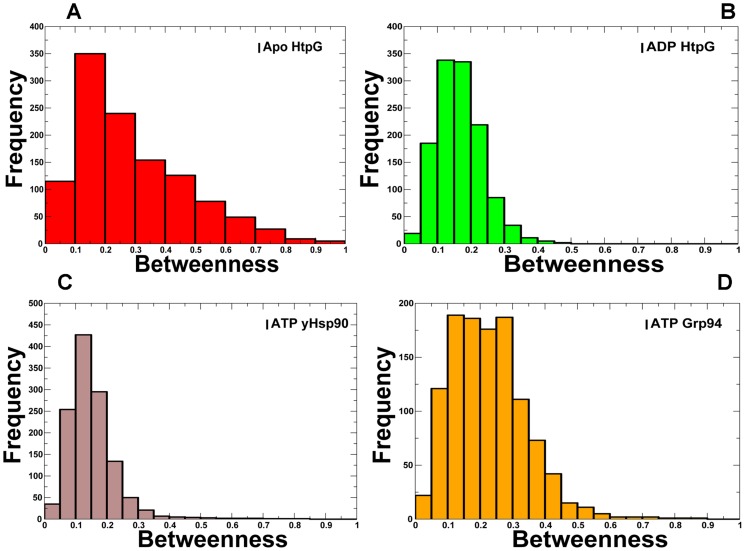
The Frequency Distributions of the Network Centrality Parameters in Hsp90 Structures. The frequency distributions of the betweenness values are shown in different functional states of Hsp90: (A) the apo-HtpG structure (red bars); (B) the ADP-HtpG structure (green bars), (C) the yeast ATP-Hsp90 structure (brown bars), and (D) the ATP-Grp94 structure (orange bars). The residue-based betweenness distributions point to small-world organization of the interaction networks in the Hsp90 structures.

To test this thesis, we constructed the frequency distributions graphs of the betweenness values in different functional states of Hsp90. Consistent with studies of protein structures [109]–[115], the residue interaction networks in the Hsp90 structures displayed a certain bias towards a Poisson-like centrality distribution and suggested a small-world network organization (Figure 10). The centrality profile in the ATP-bound Hsp90 deviated from the random graph model, which was manifested by a sharper decay and a longer tail of the distribution. A similar effect was seen in proteins strongly affected by specific functional requirements such as thermal stability, ligand binding, and specificity of enzyme catalysis [116]. We then examined the network centrality profiles and the spatial distributions of mediating residues in different functional states of Hsp90. This analysis was undertaken to establish a relationship between structural stability, global centrality and functional significance of hotspot residues involved in chaperone regulation.

**Figure 11:**
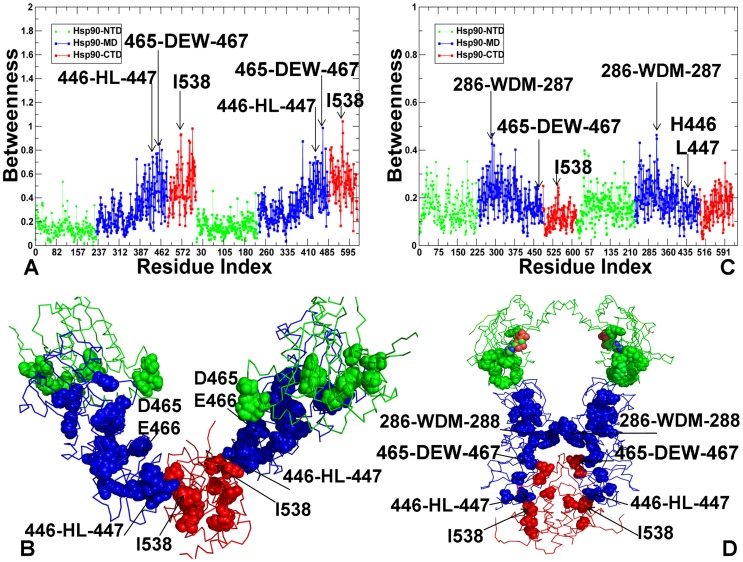
The Residue-Based Betweenness Profiles of the HtpG Structures. Dynamics-based analysis of network centrality in the HtpG crystal structures. The residue-based betweenness profiles are shown for the apo HtpG crystal structure (A) and the ADP-HtpG crystal structure (C). The betweenness profiles are shown in green for the NTD residues, in blue for the MD residues, and in red for the CTD residues. The peaks of the betweenness profiles corresponding to functionally important residues are indicated by arrows and annotated. Structural mapping of high betweenness residues that correspond to functionally important sites is shown for the apo HtpG crystal structure (B) and the ADP-HtpG crystal structure (D). The protein structures are shown in a backbone trace representation and domain-colored: NTD (in green), MD (in blue), and CTD (in red). The functional residues of high centrality are shown in spheres and colored according to their respective domains. Structural positions of high centrality functional residues are indicated by arrows.

The residues with a higher betweenness in the apo-HtpG were primarily assembled in the MD and CTD regions (Figure 11 A, B), but were distributed more uniformly in the ADP-bound HtpG (Figure 11 C, D). In the apo-HtpG crystal structures the betweenness profile revealed noticeable peaks corresponding to the important functional residues. In particular, a regulatory residue H446 can control pH-dependent conformational equilibrium of the HtpG homologue, and a single point H446K mutation could readily shift the conformational preferences of the chaperone towards the low pH state [58]. Another high betweenness residue corresponded to a regulatory switch I538 from the HtpG-CTD (A577 in yeast Hsp90) [90]. A severe effect of mutations in these regulatory sites may be partly associated with a dramatic reduction of communication flow through mutated nodes. Interestingly, the high centrality residues in the HtpG structures could form a network of small modules that are densely connected within each module, but with a fewer number of connecting nodes between different modules. The network of high centrality residues in ADP-HtpG was rather fragmented, which is consistent with structural experiments suggesting that ADP-bound form is the most solvent accessible and flexible state during functional cycle of the chaperone [62]. The high betweenness peaks in the ADP-HtpG corresponded to relatively flexible residues (286-WDMWNR-291) centered on the projecting loop (residues 281–296). The structure of this motif is invariant across all Hsp90-MD structures and the exposed hydrophobic face of this loop has been implicated a potential client protein binding site [56]. In the ADP-HtpG structure, this loop on both monomers extends into the dimer cleft, potentially providing an optimal route for the inter-monomer communication (Figure 11D). Although alterations in this loop have only minor effects on the chaperone activity, mutations in the adjacent hydrophobic patch (329-FWLF-332 in yeast Hsp90) could result in a considerable deterioration of the client recruitment function [94].

**Figure 12:**
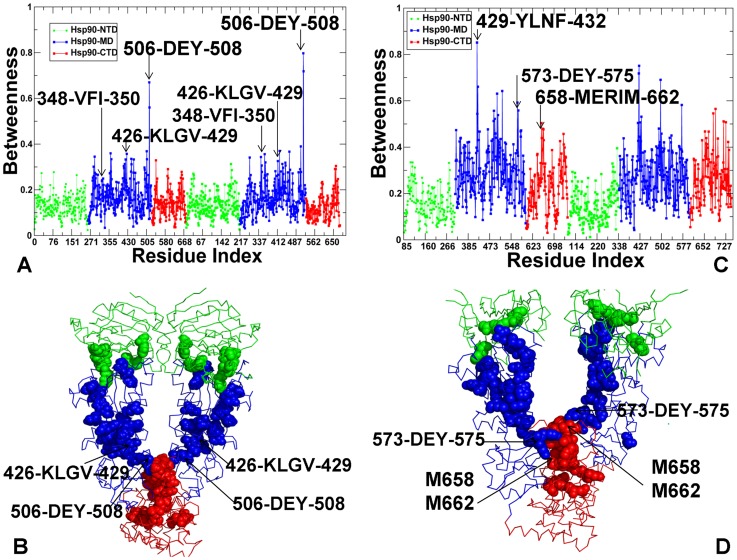
The Residue-Based Betweenness Profiles of the ATP-bound yeast Hsp90 and Grp94 Structures. The residue-based betweenness profiles are shown for the crystal structure of yeast ATP-Hsp90 (A) and the crystal structure of ATP-Grp94 (C). The betweenness profiles are shown in green for the NTD residues, in blue for the MD residues, and in red for the CTD residues. The peaks of the betweenness profiles corresponding to functionally important residues are indicated by arrows and annotated. Structural mapping of high betweenness residues that correspond to functionally important sites is shown for the crystal structure of yeast ATP-Hsp90 (B) and the crystal structure of ATP-Grp94 (D). The protein structures are shown in a backbone trace protein representation and colored according to their domain nomenclature as in Figure 11. The functional residues of high centrality are shown in spheres and colored according to their respective domains. Structural positions of high centrality residues are indicated by arrows.

The characteristic feature of the active ATP-Hsp90 dimer is the higher average betweenness of the Hsp90-MD residues and the emergence of sharp peaks for residues located at the inter-domain boundaries (Figure 12 A, B). Importantly, most of the high betweenness peaks corresponded to known functional residues responsible for regulation of the chaperone activity and the Hsp90-ATPase cycle. A central finding of this analysis is a striking similarity between functional residues corresponding to the peaks in structural stability profiles (Figures 4–6) and network centrality distributions (Figures 11,12). In particular, high betweenness residue clusters 348-VFIT-351 and 426-KLGV-429 could form key mediating sites of the inter-domain communications (Figure 12 A, B). Mutations of hydrophobic residues V348 and F349 at the NTD-MD interface could impair the ATPase activity and allosteric signaling in yeast Hsp90 [94]. Another broad peak corresponded to residues 426-KLGV-429 at the MD-CTD interface. Notably, these residues also displayed the high level of amide hydrogen protection in HX-MS experiments [62]. We found that high centrality residues could form inter-modular “edges” connecting structurally stable communities. In particular, a group of mediating residues at the MD-CTD interface (K426, E507, W585, and M589) could provide channel for rapid and robust communication at the MD-CTD interface and proper modulation of the inter-domain movements (Figure 12A, B). Interestingly, E507, W585, and Y508 residues are important for in vivo chaperone function and potentially involved in client binding [74]. The betweenness profile of the ATP-Grp94 (Figure 12 C, D) is also consistent with the force constant analysis by pointing to the lack of high centrality residues in the Grp94-NTDs. Structurally stable residues with high betweenness values are primarily assembled in the Grp94-MD and at the MD-CTD interface. The key role of the MD and CTD in maintaining structural stability of Grp94 is fully consistent with the experimental data, since the truncation of the CTD residues was shown to result in a considerable loss of the ATPase activity [61]. The characteristic peaks correspond to the MD-CTD inter-domain regions and involve residue clusters 430-LNF-432, 573-DEY-575, and 658-MERIM-662. Hence, functional role of the conserved M658-M662 pair may be also associated with their involvement in allosteric communication between MD and CTD regions.

Collectively, these results suggested that effective allosteric communications in different functional states of the Hsp90 chaperone can be mediated by structurally stable residues that exhibit high centrality properties. In this context, the network analysis revealed that high centrality centers could be surrounded by clusters of neighboring residues with fairly high betweenness values. As a result, functional sites critical for allosteric signaling could be supported and shielded by neighboring residues that have sufficient communication capabilities to ensure the network resilience in the fluctuating protein environment. Similarly to local hubs, the high centrality residues may favor a rich-club organization that would increase network tolerance to random failures. The severity of mutational defects in functional sites may be determined by a combination of structural stability requirements and network centrality properties. Although mutations of functional residues may often result in a loss of activity, some of these changes could be tolerated and rescued by a well-connected network of the “supporting cast” residues that may assume additional functional responsibilities in the altered interaction network. According to our findings, mediating residues corresponding to common peaks in the force constant profiles and network centrality distributions could bridge structurally rigid and flexible regions. Consequently, allosteric interactions in the Hsp90 chaperone may be mediated via a mechanism that combines efficient communication via a single pathway of structurally rigid residues and robust communication via a dynamic ensemble of communication pathways that couples structurally rigid nodes and more flexible residues. The diversity of allosteric communication mechanisms could ensure a proper balance of the network efficiency and functional redundancy required to maintain resilience against random attacks in the fluctuating protein environment. The additional layers of protection in regulatory mechanisms can be provided through recruitment of cochaperones and posttranslational modifications [117], [118].

In summary, integration of biophysical simulations and structure-based network analysis has recapitulated a broad range of structural and mutagenesis experiments by revealing small-world organization of the interaction networks and strong correspondence between structurally stable inter-domain sites, global mediating residues and key functional hot spots of the Hsp90 activity. The results suggested that allosteric regulation of the Hsp90 chaperone may be mediated by modules of structurally stable residues that display high centrality in the global interaction network. Mapping of the conformational landscape with the network centrality parameters indicated that stable interaction communities and global mediating sites may act concertedly with the shifts in the conformational equilibrium and characterize the vast majority of functionally significant chaperone residues. Among interesting findings of this analysis is that common characteristic peaks in structural stability profiles and network centrality distributions may converge to the same functional hotspots of chaperone activity. We found that the nucleotide-specific rewiring of the network topology and assortative organization of critical local hubs and high centrality functional residues in yeast ATP-Hsp90 may protect the active form of the chaperone from random perturbations and detrimental mutations. These results confirmed that a relatively small number of functional residues may be utilized by the chaperone machinery as central regulators of multiple functions, including structural stability, allosteric communications, progression of the ATPase cycle, and binding with cochaperones and client proteins. Further understanding of structure, dynamics, and stability of the Hsp90 interactions in allosterically regulated complexes with client proteins and cochaperones may provide detailed clues to molecular mechanisms and guide discovery of allosteric modulators.

## Materials and Methods

### MD Simulations

MD simulations of the Hsp90 structures were performed as described in [81] for apo and ATP-bound conformations of yeast Hsp90 (PDB ID 2CG9) [55]; an apo form of the bacterial homologue HtpG (PDB ID 2IOQ), ADP-bound and ATP-bound forms of HtpG (PDB ID 2IOP) [56]; an extended open form of HtpG (solution structure [57], [58]; ADP-bound Grp94 (PDB ID 2O1V) and ATP-bound Grp94 (PDB ID 2O1U) [61]. The initial conformation used in simulations of the ATP-bound HtpG was based on the crystal structure of the ADP-HtpG complex [56]. The initial conformation of the apo form of yeast Hsp90 was obtained by removing the nucleotide from the ATP-bound yeast Hsp90 complex [55] followed by structural refinement. All crystallographic water molecules, bound inhibitors, and other heteroatoms were removed. The unresolved structural segments and disordered loops were modeled with the ModLoop server [119], [120]. The initial structures were solvated in a water box with the buffering distance of 10 Å. The system was subjected to initial minimization for 20,000 steps keeping protein backbone fixed which was followed by 20,000 steps of minimization without any constraints. MD simulations were carried out using NAMD 2.6 [121] with the CHARMM27 force field [122], [123] and the explicit TIP3P water model as implemented in NAMD 2.6 [124]. The employed MD protocol is consistent with the setup in MD simulations of the Hsp90 crystal structures and was described in details in our earlier studies [81].

For each of the HtpG, yeast Hsp90 and Grp94 structures two MD trajectories with different random starting velocities were run using 50 ns production period. Equilibration was done in stages by gradually increasing the system temperature in steps of 20 K starting from 10 K until 310 K. At each stage, 30 ps equilibration was run using a restraint of 10 Kcalmol^−1^ Å^−2^ on *C_α_* atoms. The system was then equilibrated for 300 ps at 310 K using Langevin piston (NPT) to achieve a uniform pressure. After the restrains were removed the system was equilibrated for 300 ps to prepare the system for simulation. An NPT simulation was run on the equilibrated structure for 50 ns keeping the temperature at 310 K and pressure at 1 bar using Langevin piston coupling algorithm. The van der Waals interactions were treated by using a switching function at 10 Å and reaching zero at a distance of 12 Å.

Principal component analysis (PCA) of the MD conformational ensembles was used to obtain the dynamic cross-correlation matrix between residues [125], [126]. The frames are saved every 20 ps, and a total of 100,000 frames were used to compute the correlation matrices for each simulation. The calculations were performed using the CARMA package [127] and PCA_NEST web-based service [128]. The cross-correlation matrix elements *C_ij_* can vary between −1 and 1. When *C_ij_* = 1, then the residues *i* and *j* are positively correlated during the course of simulation and concertedly move at the same direction, while if *C_ij_* = −1 the residues *i* and *j* are anti-correlated. Residues that move independently of one another or in perpendicular directions have a correlation value close to zero.

### Structural Stability Analysis

We employed the dynamics-based analysis of structural stability [88], [89] by analyzing the mechanical properties of the Hsp90 chaperone in different functional states. In this approach, the displacement of each residue with respect to the rest of the protein structure is monitored during the course of MD simulation. The fluctuations of the mean distance between a given residue and all other residues in the protein structure are evaluated by computing the force constant profile that measures the energy cost of displacing a given residue during conformational equilibrium changes. The force constant parameters can be obtained by perturbing the mean distance using constrained energy minimization [88] or from the fluctuations of the mean distance in MD simulations [89]. This approach can adequately describe protein mechanics at the residue level and characterize structurally stable protein regions [129],[130]. All-atom MD simulations and elastic network models using a coarse-grained protein representation typically converge to similar force constant profiles and can capture the global dynamics properties for a variety of protein structures [131]. In our study, MD simulations of the Hsp90 structures are analyzed by computing the fluctuations of the mean distance between each atom within a given residue and the atoms that belong to the remaining residues of the protein. The force constant for each residue is computed as the average of the force constants for all its atoms *i*. Alternatively, the mean fluctuations of a given residue can be also characterized using only *C_α_* atom positions. In our model, the force constant for each residue is calculated by averaging the distances between the residues over the MD trajectory using the following expression:
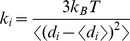
(1)

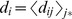
(2)where *d_ij_* is the instantaneous distance between residue *i* and residue *j*, *k_B_* is the Boltzmann constant, *T* = 300 K. 〈〉 denotes an average taken over the MD simulation trajectory and 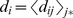 is the average distance from residue *i* to all other atoms *j* in the protein (the sum over 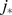 implies the exclusion of the atoms that belong to the residue *i*). The interactions between the *C_α_* atom of residue *i* and the *C_α_* atom of the neighboring residues *i*−1 and *i*+1 are excluded in the calculation since the corresponding distances are nearly constant. The inverse of these fluctuations yields an effective force constant *k_i_* that describes the ease of moving an atom with respect to the protein structure. The residue-based force constant profiles are used to characterize structural stability and conformational flexibility of protein residues.

### Protein Structure Network Analysis

The network analysis of the Hsp90 structures was conducted by generating graphs in which amino acid residues were considered as nodes connected by edges corresponding to the noncovalent interactions. The details of the construction of such a graph at a particular interaction cut-off (*I*_min_) were extensively discussed [39], [40].

Here, we describe the main steps in the construction of protein structure networks adopted in our study. The interactions between side chain atoms of amino acid residues (nodes) define edges of the protein structure network and are evaluated from the normalized number of contacts between nodes. The non-covalent interactions between sequence neighbors are ignored in the graph construction. The interaction between two residues *i* and *j* is measured as
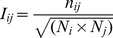
(3)In the original formulation of the graph construction procedure [39], [40], the interaction parameter was also defined as a percentage given by:
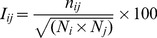
(4)where *n_ij_* is number of distinct atom pairs between the side chains of amino acid residues *i* and *j* that lie within a distance of 4.5 Å. *N_i_* and *N_j_* are the normalization factors for residues *i* and *j* as determined in [39]. *I_ij_* is evaluated for all pairs (*i*, *j*) in the protein structure. The pair of residues with the interaction *I_ij_* greater than a user-defined cut-off (*I*_min_) are connected by edges and produce a protein structure network graph for a given interaction cutoff *I*_min_. According to the analysis of a large number of protein structures, *I*_min_ values could vary from 1% to 15%, where the lower *I*_min_, the higher is the graph connectivity. The optimal interaction cutoff, that can produce adequate graph representations for a wide range of protein structures, was determined as the transition point for the largest noncovalently connected cluster [40]. According to this definition, the *I*_min_ value _typically_ lies in the range 2–4% for a diverse spectrum of protein systems and molecular complexes [39]–[44]. The number of residue-residue interactions typically drastically reduces above these *I*_min_ values, producing an adequate graph representation of residue-residue interactions in protein structures [39], [40]. A similar analysis was conducted in our study. In the graph-based analysis of the Hsp90 structures, at *I*_min_ = 1%, all residue nodes are connected by edges, while at *I*_min_ = 10%, there are typically very few residue nodes connected by non-covalent edges (interactions). We found that the appropriate transition value for the cut-off *I*_min_ = 2.5%–3%. Hence, in the present study, any pair of residues are connected in the protein structure graph if *I_ij_*>*I*_min_ = 3.0%.

The analysis of the interaction networks was done using network parameters such as hubs, cliques and communities. The hubs are highly connected nodes in the network. If the total number of edges incident on the node (called the degree of a node) is at least 4 the node is identified as a hub. The *k*-cliques are complete sub graphs of size *k* in which each node is connected to every other node. In our application, a *k*-clique is defined as a set of *k* nodes that are represented by the protein residues in which each node is connected to all the other nodes. A *k*-clique community is determined by the Clique Percolation Method [132] as a subgraph containing *k*-cliques that can be reached from each other through a series of adjacent *k*-cliques. We have used a community definition according to which in a *k*-clique community two *k*-cliques share *k*−1 or *k*−2 nodes. The construction of protein structure graphs was done with the web-based tool that converts protein structures into graphs (http://vishgraph.mbu.iisc.ernet.in/GraProStr/). Computation of the network parameters was performed using the Clique Percolation Method as implemented in the CFinder program [133]. Given the chosen interaction cutoff *I*_min_ we typically obtain communities formed as a union of *k* = 3 and *k* = 4 cliques.

A weighted network representation of the protein structure described in [45] is also adopted in this study. This model incorporates both the non-covalent connectivity of side chains and residue cross-correlation fluctuation matrix in the construction of network graphs. In this dynamic protein network, the weight *w_ij_*of an edge between nodes *i* and *j* is determined by the extent of dynamic information flow through that edge as measured by the correlation between respective residues. The weight *w_ij_* is defined according to [45] as 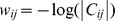 where *C_ij_* is the element of the covariance matrix measuring the cross-correlation between fluctuations of residues is *i* and *j* obtained from MD simulations. The interaction cliques and communities were considered to be dynamically stable if these interaction networks remained to be intact in more than 75% of the ensemble conformations. The conformational ensemble used in the protein network analysis was obtained from MD simulations as described in [81] and included a total of 500 representative snapshots.

### Network Centrality Analysis

Using the constructed protein structure networks, we also computed the global centrality measures such as residue degree, closeness and betweenness. Central to the computation of these parameters is the determination of the shortest paths between two given residues. The length of a path 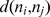 between distant nodes *n_i_* and *n_j_* is the sum of the edge weights between the consecutive nodes 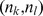 along the path:
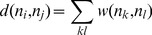
(5)The shortest paths between two residues 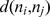 are determined using the Floyd–Warshall algorithm [134] that compares all possible paths through the graph between each pair of residue nodes. At the first step, the distance between connected residues was considered to be one, and the shortest path was identified as the path in which the two distant residues were connected by the smallest number of intermediate residues. Network graph calculations were performed using the python module Network [135]. To select the shortest paths that consist of dynamically correlated intermediate residues, we considered the short paths that included sufficiently correlated (*C_ij_* = 0.5–1.0) intermediate residues. This procedure was adopted from previous studies [45], [46] which defined an ensemble of suboptimal pathways connecting spatially separated sites based on the tolerance threshold for the edge weight of connecting residues *C_ij_* = 0.5; 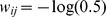 = 0.69.

The degree of a node is a centrality measure of the local connectivity in the interaction network. The degree of residue *i* is the number of its direct connections to other residues and is computed as follows:
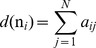
(6)where *a_ij_* is the element of adjacency matrix *A*, and *N* is the total number of nodes in the residue interaction network.

The betweenness of residue *i* is defined to be the sum of the fraction of shortest paths between all pairs of residues that pass through residue *i*. Residues with high occurrence in the shortest paths connecting all residue pairs have a higher betweenness values. The normalized betweenness of residue *i* can be expressed as follows:
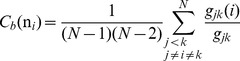
(7)where *g_jk_* is the number of shortest paths between residues *j* and *k*, and *g_jk_*(*i*) is the fraction of these shortest paths that pass through residue *i*.

## Supporting Information

Figure S1 **Equilibrium Fluctuations of the HtpG Crystal Structures.** A comparative analysis of equilibrium fluctuations for the HtpG structures. The computed B-factors are shown for the SAXS structure of apo-HtpG (in blue); the crystal structure of apo-HtpG (in red); and the crystal structure of ADP-HtpG (in green).The residue-based profiles are based on the consecutive residue numbering adopted from the original crystallographic residue annotation as described in Figure 2. For clarity of presentation, the equilibrium profiles are shown only for one monomer of the homodimer.
(TIF)

Figure S2 **The Force Constant Stability Analysis of the Grp94 Chaperone.** (A) The residue-based force constant profile of the ATP-bound Grp94 chaperone structure. The NTD residues are in green, MD residues are in blue, and CTD residues are in red. The residue-based dynamic profiles are annotated using the residue numbering in the original crystal structure [61]. The peaks of the force constant profiles corresponding to functionally important residues are indicated by arrows and annotated. Functional residues corresponding to the peaks in the force constant distribution are mapped onto the domain-colored crystal structure of the ATP-bound Grp94 (B) and onto the functional dynamics profile of the ATP-bound Grp94 (C). Functional residues are annotated and shown in spheres and colored as in Figure 4.
(TIF)

Figure S3 **Nucleotide-Specific Modulation of Solvent Accessibility in the Hsp90 Structures: The Ensemble-Based Differential Profiles.** The ensemble-based differential profiles of solvent accessibility are based on a computational procedure for calculating the depth of a residue from the protein surface [100]. The effect of nucleotide binding is evaluated using differential plots of the residue depth profiles between ATP-bound HtpG and apo-HtpG (A) and between ATP-bound yeast Hsp90 and apo-Hsp90 (B). The computed profiles are directly compared with the HX-MS experiments, showing ATP-induced protection in the key functional regions. The functional regions that experienced considerable nucleotide-specific changes in the residue depth profiles are annotated and indicated by ovals.
(TIF)

Figure S4 **Structural Alignment of the Interaction Communities with the Conformational Dynamics Profiles of Hsp90.** The network-based interaction communities are mapped onto the conformational dynamics profiles of the Hsp90 structures in the space of principal modes. Structural maps of communities are shown for the solution structure of HtpG (A), the crystal structure of apo HtpG (B), the crystal structure of ADP-HtpG (C), the crystal structure of ATP-Grp94 (D), and the crystal structure of yeast ATP-Hsp90 (E). The functional dynamics profiles are obtained using PCA of the MD-based conformational ensembles averaged over three lowest frequency modes. A ribbon protein representation is employed. The color gradient from blue to red indicates the decreasing structural stability (or increasing conformational mobility) of protein residues. The residues in the interaction communities are highlighted in spheres and colored according to their level of rigidity (flexibility) in the functional dynamics profiles. The communities are primarily aligned with the structurally rigid regions in the global dynamics profiles of the Hsp90 structures.
(TIF)

Figure S5 **The Distribution and Composition of Residue Hubs in the HtpG Structures.** The spatial distribution of local residue hubs are shown for the SAXS structure of apo-HtpG (A), and the crystal structure of apo-HtpG (B). The protein structures are shown in a ribbon representation and colored according to their domain nomenclature: NTD is in green, MD is in blue, and CTD is in red. The hub residues are shown in spheres and colored according to their respective domains. The amino acid composition of highly connected hub nodes is shown for the SAXS structure in blue bars (C), and for the crystal structure of apo-HtpG structure in red bars (D). The residue hubs with the number of connected residues exceeding the threshold of four are shown.
(TIF)

Figure S6 **The Distribution and Composition of Residue Hubs in the Nucleotide-Bound Chaperone Structures.** The spatial distribution of local residue hubs is shown for the crystal structure of ADP-HtpG (A), ATP-Hsp90 (B), and ATP-Grp94 (C). The protein structures are shown in a ribbon representation and colored according to their domain nomenclature: NTD is in green, MD is in blue, and CTD is in red. The hub residues are shown in spheres and colored according to their respective domains. The amino acid composition of highly connected hub nodes is shown for the ADP-HtpG structure in green bars (D), for the ATP-Hsp90 structure in brown bars (E), and for the ATP-Grp94 in orange bars (F). The residue hubs with the number of connected residues exceeding the threshold of four are shown.
(TIF)
