# Supplementary figures and images for: Computational Modeling of Allosteric Regulation in the Hsp90 Chaperones: A Statistical Ensemble Analysis of Protein Structure Networks and Allosteric Communications
Source: PLoS Comput Biol. 2014 Jun 12;10(6):e1003679. doi: 10.1371/journal.pcbi.1003679 (PMC4055421; doi:10.1371/journal.pcbi.1003679)

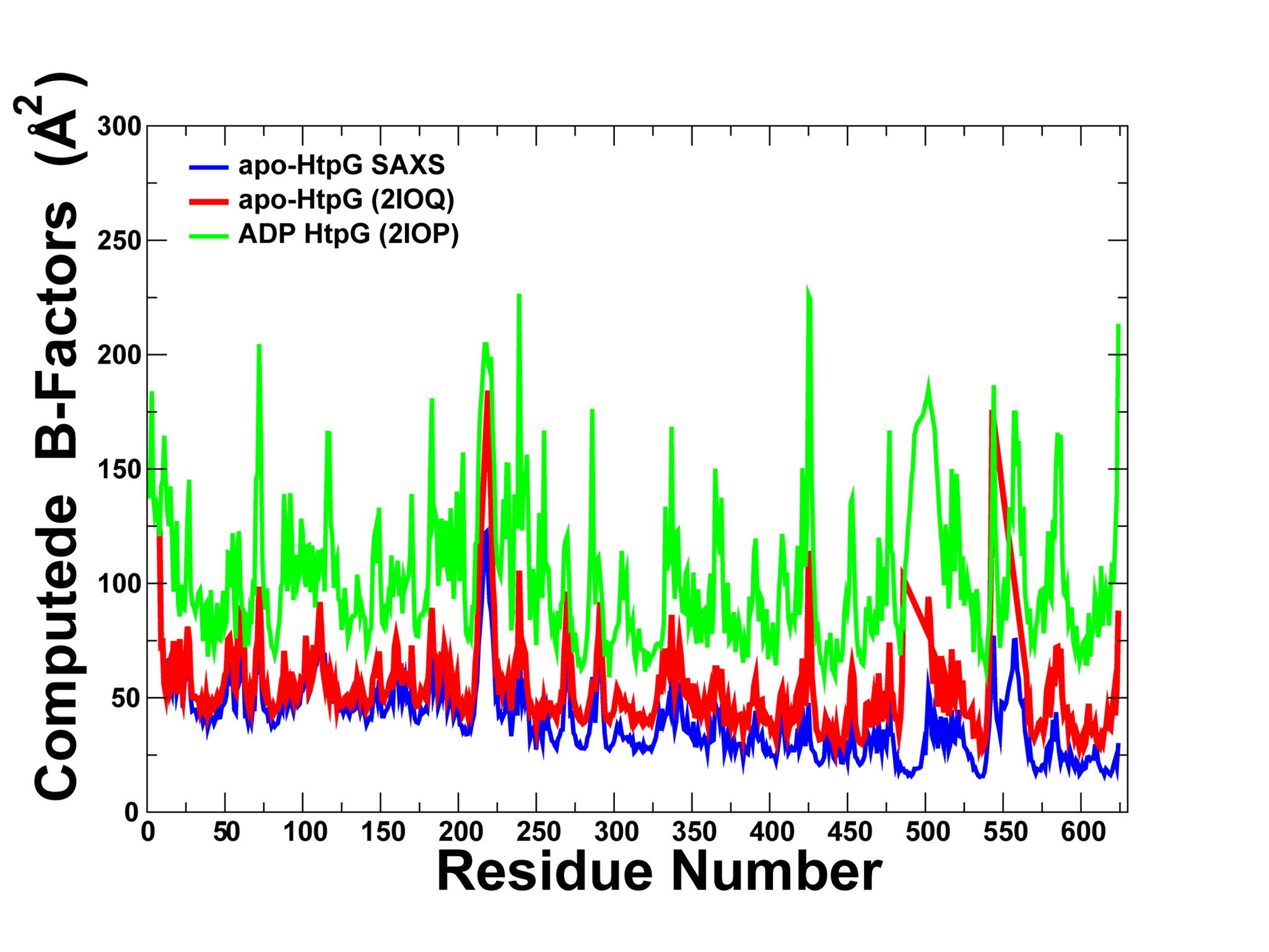

Supplement: Figure S1 — Equilibrium Fluctuations of the HtpG Crystal Structures. A comparative analysis of equilibrium fluctuations for the HtpG structures. The computed B-factors are shown for the SAXS structure of apo-HtpG (in blue); the crystal structure of apo-HtpG (in red); and the crystal structure of ADP-HtpG (in green).The residue-based profiles are based on the consecutive residue numbering adopted from the original crystallographic residue annotation as described in Figure 2. For clarity of presentation, the equilibrium profiles are shown only for one monomer of the homodimer. (TIF) [file pcbi.1003679.s001.tif]

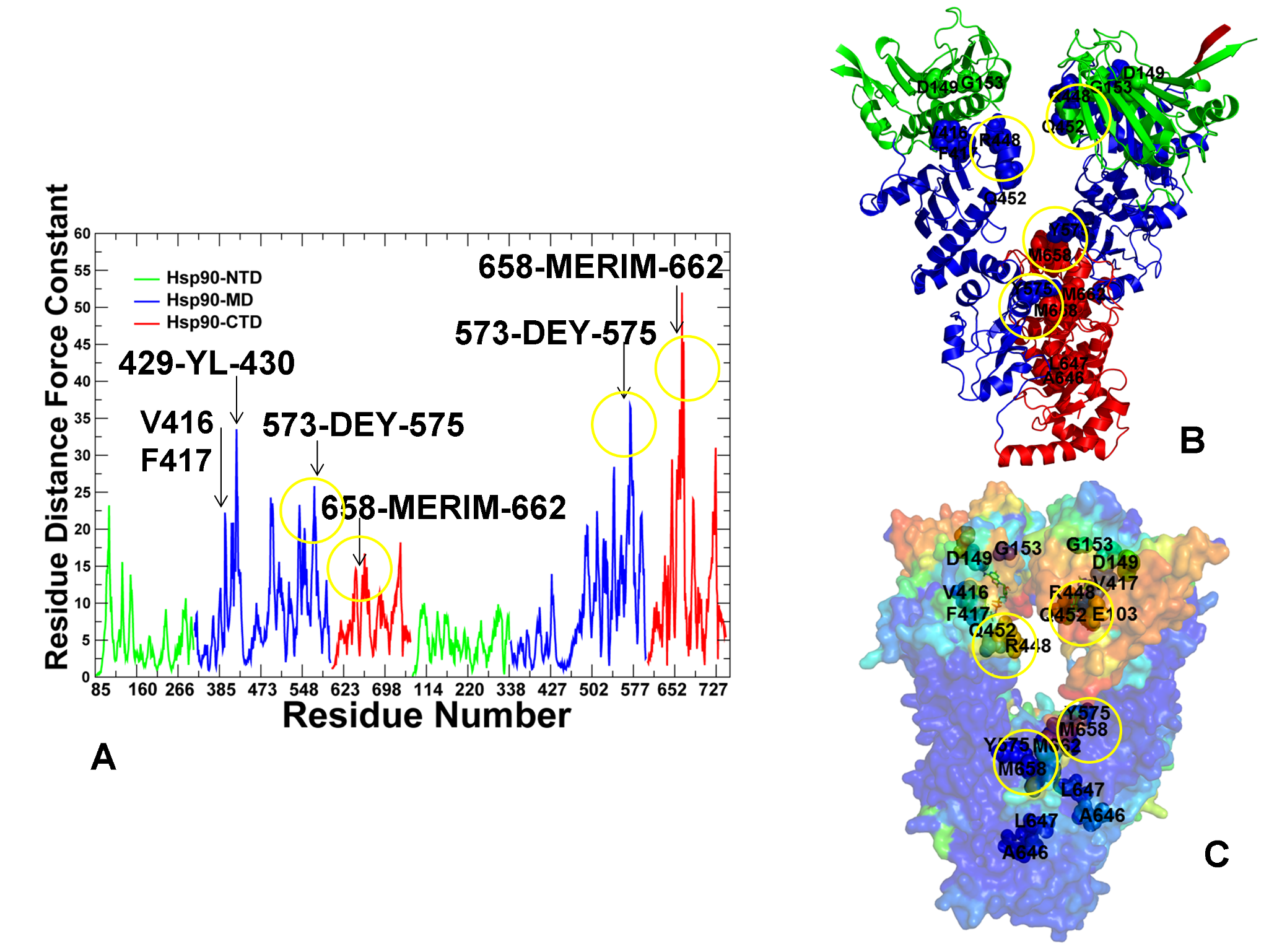

Supplement: Figure S2 — The Force Constant Stability Analysis of the Grp94 Chaperone. (A) The residue-based force constant profile of the ATP-bound Grp94 chaperone structure. The NTD residues are in green, MD residues are in blue, and CTD residues are in red. The residue-based dynamic profiles are annotated using the residue numbering in the original crystal structure [61]. The peaks of the force constant profiles corresponding to functionally important residues are indicated by arrows and annotated. Functional residues corresponding to the peaks in the force constant distribution are mapped onto the domain-colored crystal structure of the ATP-bound Grp94 (B) and onto the functional dynamics profile of the ATP-bound Grp94 (C). Functional residues are annotated and shown in spheres and colored as in Figure 4. (TIF) [file pcbi.1003679.s002.tif]

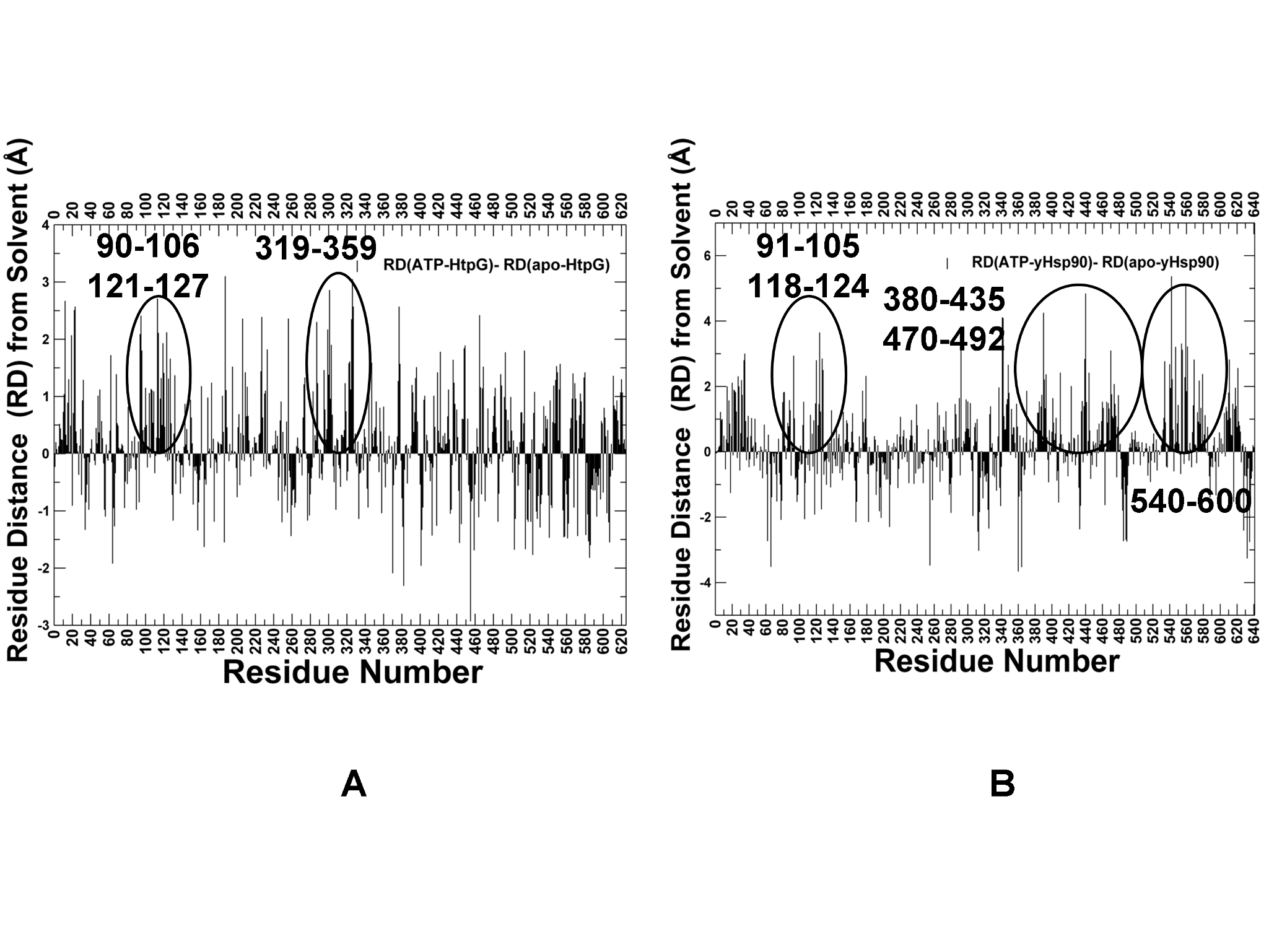

Supplement: Figure S3 — Nucleotide-Specific Modulation of Solvent Accessibility in the Hsp90 Structures: The Ensemble-Based Differential Profiles. The ensemble-based differential profiles of solvent accessibility are based on a computational procedure for calculating the depth of a residue from the protein surface [100]. The effect of nucleotide binding is evaluated using differential plots of the residue depth profiles between ATP-bound HtpG and apo-HtpG (A) and between ATP-bound yeast Hsp90 and apo-Hsp90 (B). The computed profiles are directly compared with the HX-MS experiments, showing ATP-induced protection in the key functional regions. The functional regions that experienced considerable nucleotide-specific changes in the residue depth profiles are annotated and indicated by ovals. (TIF) [file pcbi.1003679.s003.tif]

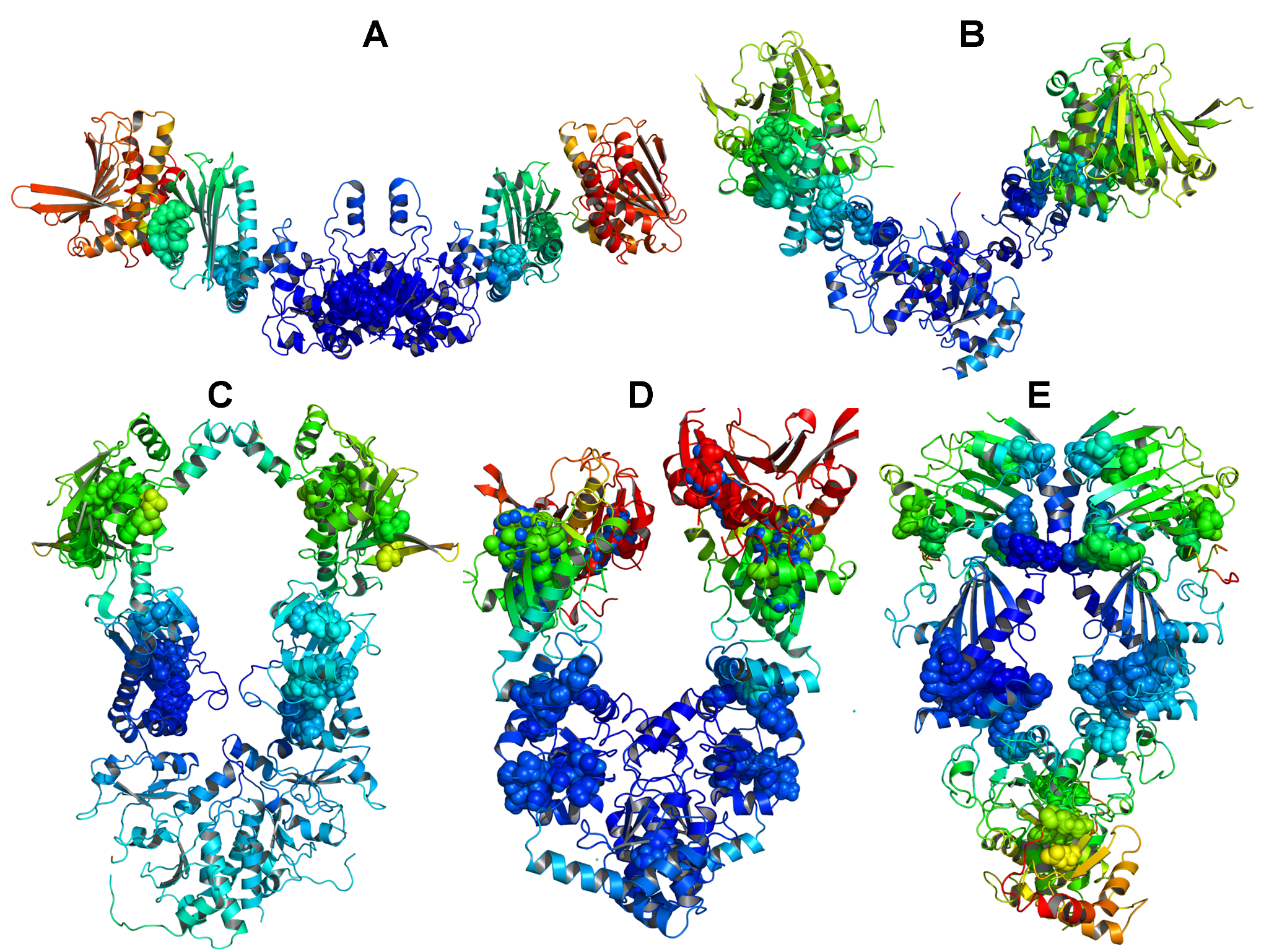

Supplement: Figure S4 — Structural Alignment of the Interaction Communities with the Conformational Dynamics Profiles of Hsp90. The network-based interaction communities are mapped onto the conformational dynamics profiles of the Hsp90 structures in the space of principal modes. Structural maps of communities are shown for the solution structure of HtpG (A), the crystal structure of apo HtpG (B), the crystal structure of ADP-HtpG (C), the crystal structure of ATP-Grp94 (D), and the crystal structure of yeast ATP-Hsp90 (E). The functional dynamics profiles are obtained using PCA of the MD-based conformational ensembles averaged over three lowest frequency modes. A ribbon protein representation is employed. The color gradient from blue to red indicates the decreasing structural stability (or increasing conformational mobility) of protein residues. The residues in the interaction communities are highlighted in spheres and colored according to their level of rigidity (flexibility) in the functional dynamics profiles. The communities are primarily aligned with the structurally rigid regions in the global dynamics profiles of the Hsp90 structures. (TIF) [file pcbi.1003679.s004.tif]

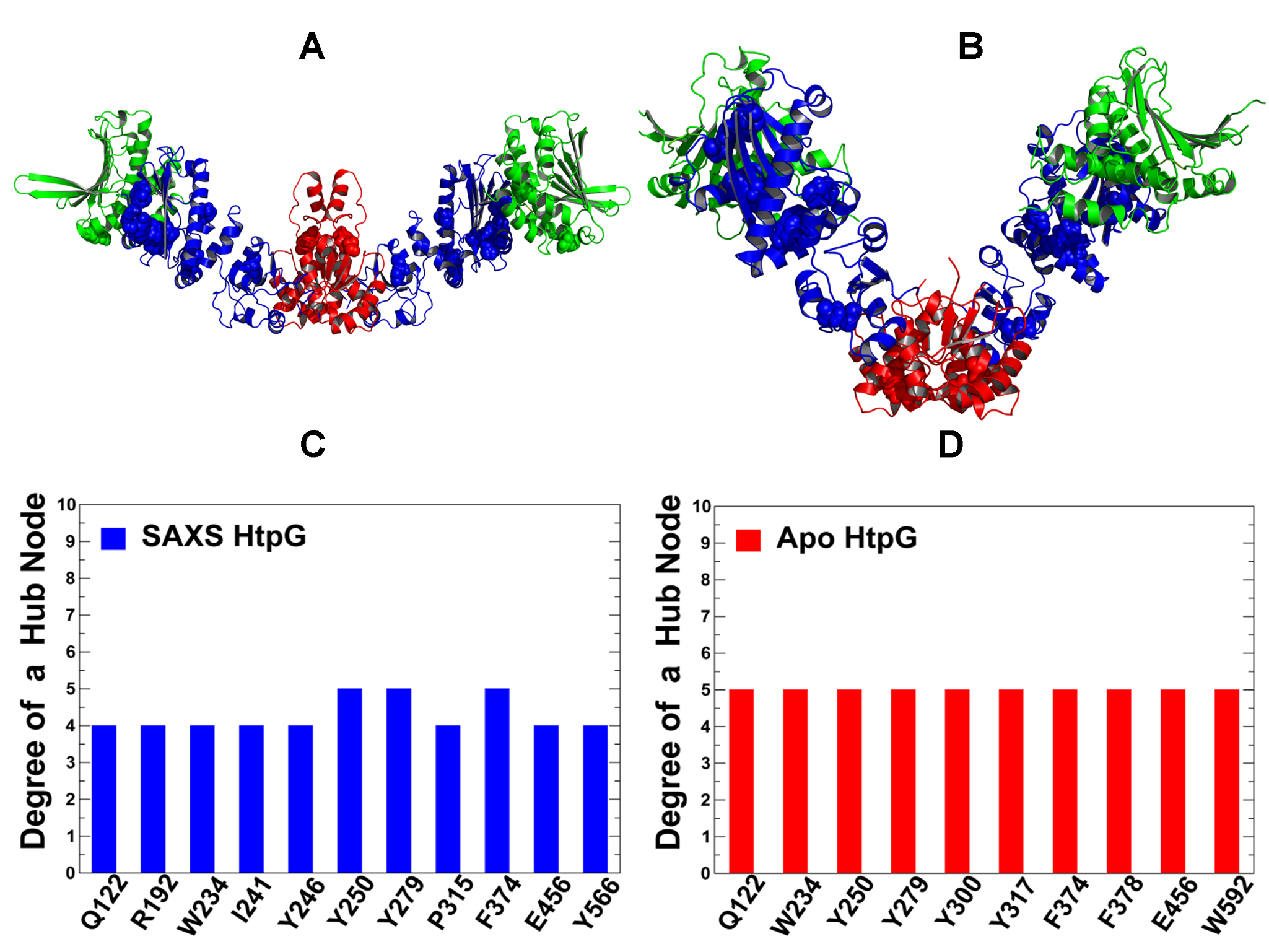

Supplement: Figure S5 — The Distribution and Composition of Residue Hubs in the HtpG Structures. The spatial distribution of local residue hubs are shown for the SAXS structure of apo-HtpG (A), and the crystal structure of apo-HtpG (B). The protein structures are shown in a ribbon representation and colored according to their domain nomenclature: NTD is in green, MD is in blue, and CTD is in red. The hub residues are shown in spheres and colored according to their respective domains. The amino acid composition of highly connected hub nodes is shown for the SAXS structure in blue bars (C), and for the crystal structure of apo-HtpG structure in red bars (D). The residue hubs with the number of connected residues exceeding the threshold of four are shown. (TIF) [file pcbi.1003679.s005.tif]

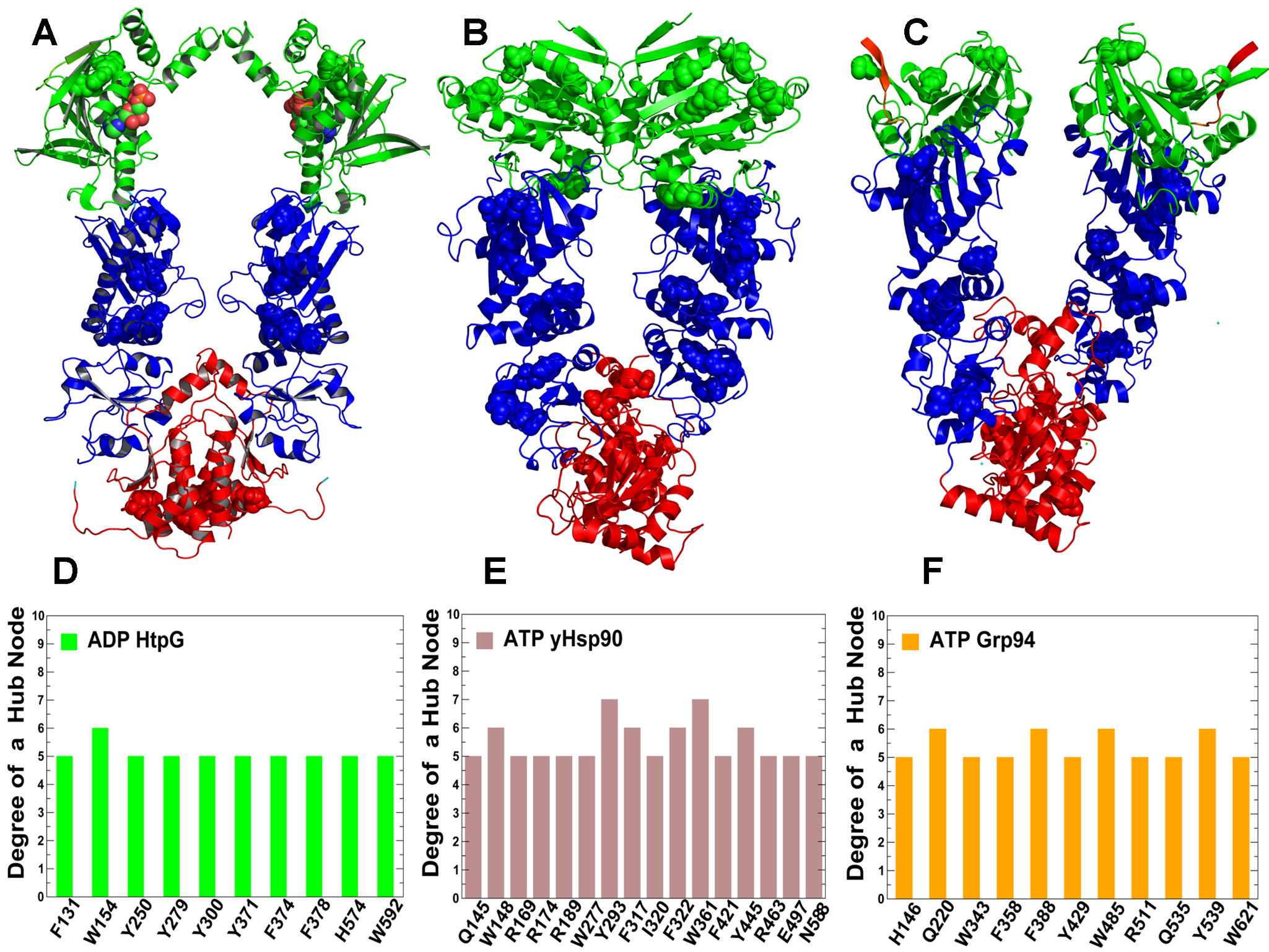

Supplement: Figure S6 — The Distribution and Composition of Residue Hubs in the Nucleotide-Bound Chaperone Structures. The spatial distribution of local residue hubs is shown for the crystal structure of ADP-HtpG (A), ATP-Hsp90 (B), and ATP-Grp94 (C). The protein structures are shown in a ribbon representation and colored according to their domain nomenclature: NTD is in green, MD is in blue, and CTD is in red. The hub residues are shown in spheres and colored according to their respective domains. The amino acid composition of highly connected hub nodes is shown for the ADP-HtpG structure in green bars (D), for the ATP-Hsp90 structure in brown bars (E), and for the ATP-Grp94 in orange bars (F). The residue hubs with the number of connected residues exceeding the threshold of four are shown. (TIF) [file pcbi.1003679.s006.tif]
